# PRISM-Seq: An Ultra-sensitive Sequencing Approach For Mapping Lentiviral Integration Sites

**DOI:** 10.64898/2025.12.20.695659

**Published:** 2025-12-23

**Authors:** VK PAL, M CANIS, E STONE, N BOARD, K LENART, M FUMAGALLI, C KOVACS, RB JONES, M NUSSENZWEIG, T HATZIIOANNOU, F MUECKSCH, PD BIENIASZ, GQ LEE

**Affiliations:** 1 Laboratory of Retrovirology, The Rockefeller University, New York.; 2 Infectious Diseases Division, Department of Medicine, Weill Cornell Medical College, New York.; 3 Laboratory of Molecular Immunology, The Rockefeller University, New York, NY 10065, USA.; 4 Maple Leaf Medical Clinic, Toronto, Ontario, Canada; 5 Department of Infectious Diseases, Virology, Heidelberg University, Heidelberg, Germany.; 6 Howard Hughes Medical Institute, The Rockefeller University, New York.

## Abstract

Retroviral integration into host genomes underlies both HIV-1 persistence and the safety and function of lentiviral vectors used in gene and cell therapies. Existing integration site assays remain limited by sensitivity, input requirements, and analytical complexity, and none have been validated at the single-molecule detection limit. Here we introduce PRISM-seq, an ultra-sensitive workflow for genome-wide recovery of lentiviral-host junctions, paired with BulkIntSiteR, an open-source, fully automated pipeline for integration site annotation. We show that PRISM-seq accurately identifies proviral insertions across diverse genomic contexts, including euchromatin, heterochromatin, and repeat-rich centromeric regions, and detects high-confidence integration events down to a single input template molecule. By systematically characterizing assay- and amplification-associated noise, we developed a five-step quality control framework that removes PCR and sequencing artifacts. PRISM-seq also enables quantitative clonal tracking through replicate-based sampling and achieves performance comparable to or exceeding high-input assays at substantially reduced cost.

## Introduction

Integration of retroviral DNA into host DNA is central to both HIV-1 pathogenesis and the use of lentiviral vectors in gene therapy. In HIV-1 infection, the site of viral integration predicts viral transcription and reactivation potential^1–3^, while in lentiviral vector-based gene therapies, vector insertional profiles inform safety and clonal dynamics^4–10^.

Existing proviral integration site sequencing assays and bioinformatics workflows (e.g., INSPIIRED^11,12^, VISPA/VISPA2^13^, QuickMap^14^, VISA^15^, MAVRIC^16^, GeIST^17^, IS-Seq^18^, VSeq-Toolkit^19^ face three practical barriers. First, many require relatively high template input, typically ~1–10 μg of genomic DNA per library^20,21^, are incompatible with single cell sorting, and none were validated down to the single-molecule detection limit nor across distinct genome contexts, including difficult-to-map regions. Second, computational workflows are fragmented, platform-specific, and/or require bioinformatics expertise for read processing and annotation^12,20^. Third, per-sample per-replicate costs remain elevated due to multi-step library construction and sequencing depth requirements.

Here, we present proviral integration site mapping sequencing (PRISM-seq), an ultra-sensitive, single-copy, and bulk-input compatible lentiviral integration site sequencing assay, together with BulkIntSiteR, a fully automated and platform-agnostic bioinformatics analysis pipeline that converts with one-click raw sequencing data files into annotated proviral integration sites.

## Results

### PRISM-seq design

**Figure 1.**
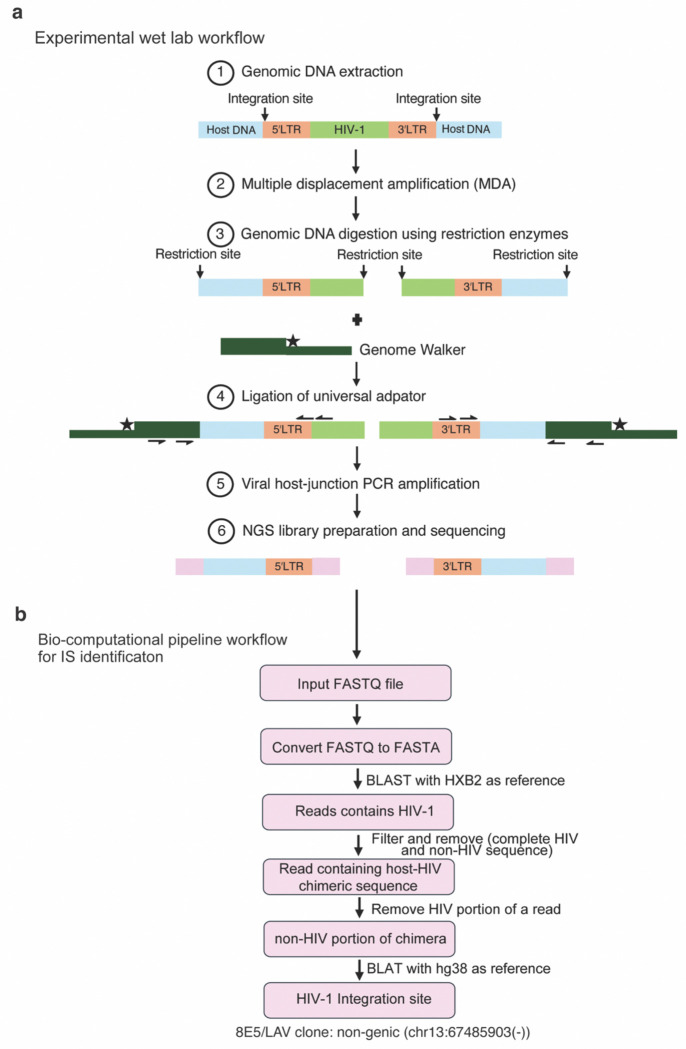
Experimental and bioinformatic workflow for proviral integration sites identification using PRISM-seq. **(a)** Experimental workflow of PRISM-seq. The viral-host junction is specifically amplified using the gDNA samples of HIV-1 infected or lentiviral vector-transduced cell samples and sequenced. **(b)** The bioinformatics steps involved in the BulkIntSiteR pipeline to process the raw sequencing data and generate an output containing the list of proviral integration sites. BulkIntSiteR normalizes junction coordinates to the 5′ LTR convention, accounting for the characteristic 4-bp offset between the 5′ and 3′ LTR junctions (Craigie and Bushman, 2012). The large disparity in junction-containing reads between the 5′ and 3′ LTR integration sites libraries (e.g. 8E5/LAV benchmarking experiment) likely reflects the differences in amplicon composition, relative to Illumina tagmentation and read length. The 5′ LTR amplicon contains ~681 nt of viral sequence, whereas the 3′ LTR amplicon contains only ~34 nt. Because tagmentation generates fragments of ~300 bp on average and the sequencing read length was 150 bp, most 5′ LTR reads fall entirely within the viral portion of the amplicon and never reach the host sequence, which would markedly reducing the number of recoverable viral-host chimeras.

PRISM-seq detects single-molecule inputs and single-copy species in bulk samples. The core of the PRISM-seq design that enabled ultra-sensitive single-molecule compatible detection is bacteriophage phi29-mediated multiple displacement amplification (MDA), which is not commonly used in integration site identification (Fig. 1a and see methods for the origin and foundation assays for PRISM-seq). In this study, reactions were performed using 50-template inputs, but the assay also accommodates substantially higher input levels (~10,000 templates^22^). MDA-amplified DNA is processed by ligation-mediated PCR (LM-PCR) involving restriction digestion, adaptor ligation, and amplification of both 5′ and 3′ virus-host junctions using virus- and adaptor-specific primer pairs (Fig. 1a, Fig. S1 and S2a). The PRISM-seq primer sets were designed for broad compatibility across lentiviruses, including HIV and lentiviral vector delivery systems (Fig. S2b)^23–27^. PRISM-seq amplicons containing viral-host junctions can be sequenced on any next-generation sequencing (NGS) platform. Reads were processed using BulkIntSiteR, a one-click, open-source computational pipeline developed for automated mapping and annotation of integration sites (Fig. 1b).

### Identification of proviral integration sites across the diverse human genome landscape

We first benchmarked PRISM-seq/BulkIntSiteR using the 8E5/LAV cell line, which harbors a single HIV-1 provirus at a known intergenic locus^28^. We identified a single viral integration site on chromosome 13 at position 67,485,907 (5’LTR) and 67,485,903 (3’LTR) in the negative strand, consistent with published data^28^. We did not observe any low-abundance secondary IS (noise).

The single provirus in 8E5/LAV cells is associated with heterochromatin (supplementary table 1). However, viral integration can occur in diverse regions of the human genome, including actively transcribing genes^29^, and in repeat-rich, telomeric, and centromeric satellite regions^3,6,30,31^. We generated 14 putative singly-infected Jurkat cell clones (Clones #1–14) using low-MOI (0.3) infection with a replication-deficient HIV-1 reporter (V1/SBP-GFP), followed by sorting of GFP/SBP-positive cells, limiting dilution, and expansion via culturing. Proviral integration sites in these 14 clones were expected to be mostly in genes since HIV-1 integration favors actively transcribing genomic regions^29^. To evaluate PRISM-seq performance within centromeric regions typically associated with difficult-to-map satellite DNA repeats, we analyzed two additional HIV-1-infected primary cell clones (#15 and #16) previously found to each contain a single provirus within centromeric regions^31^.

Without noise removal, our raw data showed 1–70 (min-max) unique proviral insertion sites in clones #1–16 (Fig. S3a-c and S4a-c). The observation that some clones contained more than one unique integration site suggested that methods for integration site determination should be systematically evaluated for assay noise, for example, by leveraging the theoretically clonal nature of our sample set.

An initial quality control filter designated each of our raw integration sites as either high- or low-confidence. High-confidence was defined if an integration site (i) contained precise viral DNA 5′ or 3′ ends linked to a human sequence without gaps/missing bases, and (ii) was detected by both 5′ LTR and 3′ LTR viral-host junctions or (iii) had greater than 10 reads when identified only by either 5′ LTR or 3′ LTR. All other integration sites were designated “low-confidence.”

Among clones #1–14, 9/14 (64%) yielded a single high-confidence integration site (Fig. S3c). Clone 6 and clone 10 showed multiple high-confidence integration sites, consistent with expectations from Poisson statistics for infections at MOI 0.3^32^. Clone 8, clone 11, and clone 12 had more than one integrations, but all were found in the same gene, suggesting potential assay-associated artifacts. We used two human reference genomes: hg38 and T2T-CHM13^33^, and their results were 100% concordant for clones #1–14. For clones 15 and 16, our data showed multiple high-confidence integration sites with proviruses integrated within the centromeric repeat regions of chromosomes 9 (8 sites) and 22 (2 sites), respectively (Fig. S4c), which were only detected using T2T-CHM13 as the reference genome, while hg38 returned no maps. Due to the clonal nature of these clones, we suspected repeat-driven ambiguity in mapping. All ambiguities were further explored in the next section.

Across all 16 analyzed clones, high-confidence proviral integration sites were distributed within 12 chromosomes spanning genic, intergenic, and repeat-rich centromeric satellite regions, confirmed by the chromHMM model^34^ based on distinct histone-mark ChIP-seq datasets from the ENCODE database^35^ (Supplementary Table 1). The average distance between proviral integration sites and the nearest accessible chromatin region (ATAC-seq peak) varied widely, ranging from 292 bp to 278,541 bp (Supplementary Table 1). These findings reflect the diverse genomic landscape of proviral integration recovered by PRISM-seq.

### Analyses of low-confidence integration sites and implementation of additional quality control filtering criteria

In 15 of 16 analyzed clones, we detected 174 low-confidence secondary integration sites. We next characterize this putative noise to design a quality-control filter. Of these low-confidence integration sites, 57/174 (33%) mapped to the same gene or within ±10 kb relative to the corresponding high-confidence integration sites (Fig. S3a-c, S4a-c, S5a-b, and supplementary table 2). Given the clonal nature of most of the samples, we reasoned they were likely artifacts. The remaining low-confidence sites were either in intergenic regions or in distinct genes.

To further investigate, we performed manual alignment of the host sequences of low-confidence integration sites, and showed that many differed from the corresponding high-confidence site only by short indels or 1–10 bp shifts in the junction sequence (e.g., clones 1, 2, 4, 8, 9, 10, 11, 13, 15, and 16) (Fig. S6-S12). These cases are likely the artifact signatures previously highlighted to be found in integration site sequencing assays, mainly because of PCR recombination, whole-genome amplification artifacts, sequencing errors, or ambiguous mapping in repeat-rich chromosomal regions^20,36,37^. Collectively, the short indel cases, same gene integration in a putative clone, and low read-count (≤10) when captured by only one proviral end accounted for 174/174 (100%) of the low-confidence integration sites detected among clones #1–16 (supplementary table 2).

Given these observations, we implemented a five-step quality control filter strategy to improve the specificity of PRISM-seq (Fig. S13). Steps 1–4 are fully automated, and the accompanying script is included with the BulkIntSiteR package; Step 5 requires manual curation and removes 5.7% (10/174) of the noise in our data. Step 5 is especially critical when proviruses are integrated within repeat-rich genomic regions: without it, 7 of 8 (87.5%) and 1 of 2 (50%) integration sites were called as high-confidence sites for the two clones (#15 and #16) with centromeric proviral insertion site (Fig. S14a-e). Incorporating step 5 yielded a single high-confidence integration site for each clone. All integration sites that passed the final filter are designated “high confidence integration sites” and used for downstream analysis.

### PRISM-seq detection efficiency and cumulative recovery in an artificial mix

**Figure 2.**
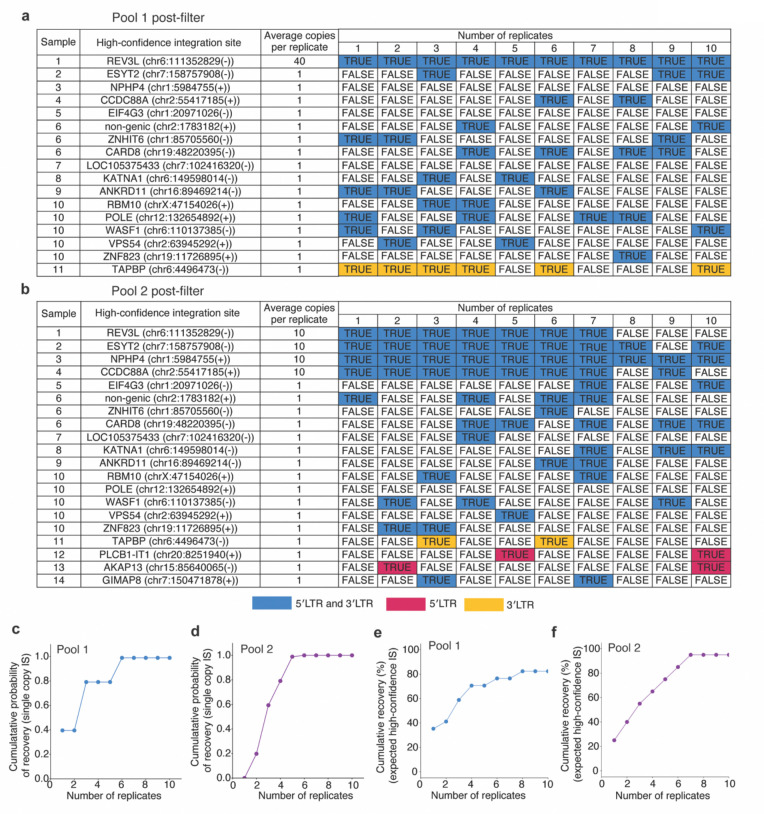
Single-molecule detection efficiency of PRISM-seq in artificial mixes. **(a and b)** Two artificial pools were constructed to test PRISM-seq’s ability to recover integration sites in samples with multiple unique integration sites at varying relative abundance. Pool 1 contained 11 of the 14 clones #1–14 encompassing at least 17 unique high confidence integration sites, whereas pool 2 contained all 14 clones #1–14 with at least 20 unique high confidence integration sites (designs described in Methods). To model differences in template clonal abundance, pool 1 included one dominant clone (Clone #1) at ~400 genome copies and ten minor clones at 10 copies each; pool 2 included four abundant clones at 100 copies each and ten minor clones at 10 copies each. Each pool was divided into ten independent genomic DNA sampling replicates resulting in each reaction containing an average of ~50 total proviral copies per reaction, equivalent to an average of ~40 copies of the dominant clone plus ten single-copy clones for pool 1, and four 10-copy clones plus ten single-copy clones for pool 2. Each of the 10 replicates was independently subjected to PRIMS-seq. The Table shows the number of high-confidence integration sites present in the artificial pool 1 (a) and pool 2 (b), respectively. The colored matrix shows the recovery of expected high-confidence integration sites across 10 replicates. The color highlights if a high-confidence integration site was recovered by both 5′LTR and 3′LTR (blue), or exclusively by either 5′LTR (red), or 3′LTR (yellow) viral-host junction. The true/false denotes if a high-confidence integration site was recovered by either proviral end. **(c and d)** Cumulative recovery of single-copy clone integration sites after adjusting for Poisson sampling expectation across 10 replicates. Clones #6 and #10 were excluded from this analysis. **(e and f)** Percent cumulative recovery of all expected high-confidence integration sites from pool 1 and pool 2, respectively.

Next, we examined whether PRISM-seq can accurately detect integration sites in samples with more than one unique integration site, each with varying abundance. Variation in template input copies, as well as multiple authentic proviral insertions, can limit detection efficiency and accuracy due to PCR competition and recombination^12,20,38^. To model this scenario, we generated two artificial pools composed of clones #1–14 (Pool 1 and Pool 2), each with integration sites represented at different relative input frequencies, and used them to assess the detection efficiency of PRISM-seq in a mixed template context (Fig. 2a-b).

With a 10-replicate setup, the abundant 40- and 10-copy-input integration site was detected at 100% efficiency in both pools (Fig. 2a-b and S15–17). For the single copy integration site templates, after adjusting for Poisson sampling expectations, the probability of detection of all high-confidence single copy integration sites increased with the number of replicates and reached 100% at 6 and 5 replicates in pool 1 and pool 2, respectively (Fig. 2c-d), despite the presence of a more abundant ~40 or ~10 copy template competitor. Moreover, the cumulative recovery of all expected high-confidence integration sites was proportional to the number of replicates in both pool 1 and pool 2 (Fig. 2e-f). Across 10 replicates, we recovered 14/17 (82%) and 19/20 (95%) of all expected high-confidence integration sites in pool 1 and pool 2, respectively. Overall, these findings demonstrate that PRISM-seq can reliably recover integration sites in a sample expected to contain both rare and abundant templates, with an average recovery of 89% integration sites across pools, and can detect down to a single-molecule input at a 100% detection efficiency with 5–6 replicates.

### Estimation of the extent of clonal expansion within a sample using PRISM-seq

**Figure 3.**
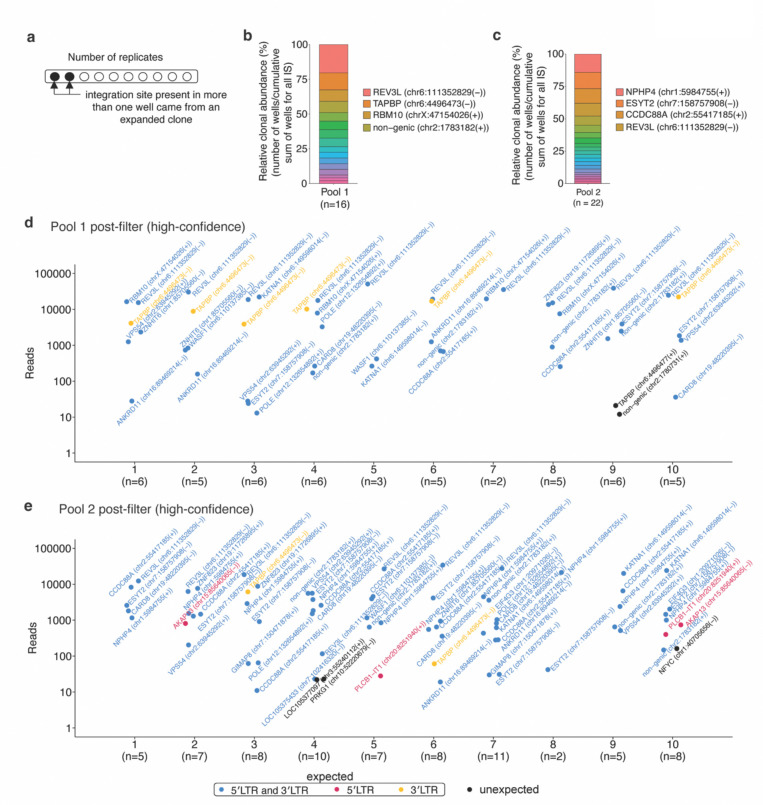
Post-quality filter recovery of high-confidence integration sites and estimation of clonal abundance. **(a)** Schematics showing the identification of expanded clones using PRISM-seq. We used the post-filtered data to evaluate integration site authenticity and to estimate clonal abundance in mixed, non-clonal samples, Pool 1 and Pool 2. The post-filtered datasets resulted in the recovery of a total of 16 high-confidence unique integration sites in pool 1 and 22 in pool 2, closely matching the expected input sites (17 for pool 1 and 20 for pool 2), with false-positive calls limited to 2 of 16 (12.5%) and 3 of 22 (13.6%), respectively. For each integration site, we counted the number of replicate reactions in which it was recovered. Integration sites detected in multiple replicates are highly likely to represent true biological events, whereas those observed only once are more likely to reflect residual noise. **(b and c)** post-filter estimation of the relative clonal abundance of individual clones found in pool 1 and pool 2 after 10 replicates, respectively. The top 4 abundant clones are labelled in the legend. **(d and e)** The dot plots show the total number of integration sites retrieved from individual sequencing replicates of pool 1 (a) and pool 2 (b), respectively. The expected high-confidence integration sites are highlighted if recovered by both 5′LTR and 3′LTR (blue), or exclusively by either 5′LTR (red), or 3′LTR (yellow) viral-host junction. The unexpected integration sites are colored in black. The quality filter led to 2 of 16 (12.5% noise) and 3 of 22 (13.6% noise) unexpected integration sites identification as noise in pool 1 and pool 2, respectively.

Across replicate analyses, repeated detection of the same integration site signifies that the original sample contains multiple copies of that viral-host junction (Fig. 3a). In a clinical context, such an observation would indicate clonal expansion of infected cells. We reasoned that the frequency with which an integration site appears across PRISM-seq replicates would be an indirect quantitative measure of the relative size of the clone harboring that integration. As proof of principle, we applied this framework to the post-filtered datasets for our artificial pools. As anticipated, the dominant clones, present at ~400 copies in pool 1 and ~100 copies in pool 2, were detected in the largest number of replicates and therefore showed the highest clonal abundance (Fig. 3b-e).

### Application to longitudinal clinical samples from a person with HIV-1 on ART

**Figure 4.**
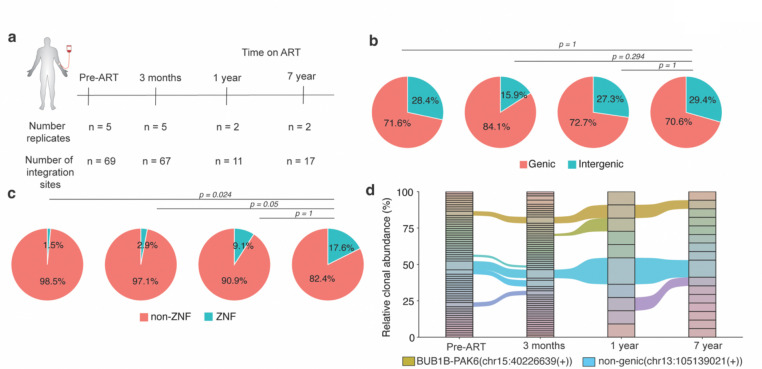
Longitudinal clonal tracking and identification of proviral integration sites in a person with HIV-1 infection. **(a)** Number of unique integration sites identified using PRISM-seq from an HIV-1 infected person under long-term ART treatment. Across the number of replicates performed, we sampled in total 250, 250, 100, and 100 HIV-containing templates at each indicated time point. **(b)** The precent genic and intergenic distribution of proviral integration sites. **(c)** The distribution of proviruses in ZNF and non-ZNF genes in the genome. P-value denotes Fisher’s exact test. **(d)** Clonal tracking of HIV-1 infected cell clones over time under ART. The shared clones identified at all the time points are labelled with their respective proviral integration sites.

To demonstrate the clinical utility of PRISM-seq, we longitudinally sampled CD4^+^ T cells from a study participant living with HIV-1 before ART initiation (pre-ART), and 3 months, 1 year, and 7 years post-ART (Supplementary Table 3). Each sample was expected to contain multiple unique integration sites originating from infected cells exhibiting variable levels of clonal expansion^39–42^. Using 2–5 replicates and after only sampling 100–250 HIV-containing templates per time point, we recovered 69, 67, 11, and 17 unique and high-confidence integration sites from each time point, respectively (Fig. 4a). The proportion of proviruses integrated into genic or intergenic regions remained stable over time (Fig. 4b). The fraction of proviruses integrated into ZNF genes increased from 2%−3% (pre-ART and 3 months) to 9% (1 year) and 18% (7 years) post-ART time points (Fig. 4c), consistent with previous reports indicating that proviruses persisting in ZNF genes and transcriptionally repressive genomic contexts are more likely to be transcriptionally silent and capable of long-term persistence^6,30^.

With longitudinal sampling and the replicate setup, we tracked clonally expanded infected cells: we observed identical viral integration sites in BUB1B-PAK6(chr15:40226639(+)) and intergenic region (chr13:105139021(+)) across all 4 time points sampled over 7 years (Fig. 4d), an indicator that HIV-1 infected cells with integrations at these loci were expanded *in vivo* and persisted during treatment over time. Overall, our findings show that PRISM-seq could be directly applied to clinical samples that had an extremely low target concentration, and provided a wealth of integration site data and biological insights with minimal sample input.

## Discussion

In this study, we present PRISM-seq and its accompanying software BulkIntSiteR. The assay recovers integration sites from single-molecule input, captures diverse genomic contexts, quantifies clonal expansion, and is compatible with *ex vivo* clinical samples.

Existing bulk integration site assays, including assays INSPIIRED^11,12^, VISPA/VISPA2^13^, QuickMap^14^, VISA^15^, MAVRIC^16^, GeIST^17^, IS-Seq^18^, VSeq-Toolkit^19^, do not support reliable single-molecule detection or broad performance across challenging genomic contexts. PRISM-seq overcomes these limitations through its MDA-enabled single-molecule capture workflow, which efficiently recovers rare viral-host junctions from minimal DNA input. Although MDA has been used previously in MIP-seq, that approach requires limiting dilution, is not designed for bulk integration site analysis, and is costly. PRISM-seq provides a bulk-input compatible, sensitive, and scalable platform for high-confidence integration site profiling.

Another key advance of PRISM-seq is the systematic characterization and mitigation of assay-associated noise, an aspect that has not been quantitatively defined in prior proviral integration site sequencing methods^12,20^ nor formalized into a standardized filtering framework. Across biologically pure samples with a single provirus integration site, we identified recurring artifact classes arising from whole-genome amplification, PCR recombination, sequencing errors, and mapping ambiguity in repeat-rich regions. These artifacts manifested as displaced junctions, small indels, low-read viral-host chimeras detected by only one LTR end, and clusters of look-alike integration sites mapping within the same gene or genomic window as the true high-confidence site. Using clonal standards, including centromere-integrated proviruses resolvable only using T2T-CHM13, we were able to distinguish genuine proviral insertions from amplification- or sequencing-derived noise and develop a five-step quality control pipeline that removes >85–90% of raw artifacts while preserving bona fide integration sites.

Application of PRISM-seq to longitudinal samples from a study participant living with HIV-1 revealed persistent clones integrated within transcriptionally repressive, ZNF-rich genomic regions. These findings reinforce prior observations that proviruses can integrate and persist in epigenetically repressed environments, including KRAB-ZNF gene clusters and other heterochromatic regions^2,6,30,43^. The compatibility of PRISM-seq with low sample input, and its ability to capture low-abundance, genomically diverse proviruses directly from clinically-derived material highlights its utility for high-resolution reservoir characterization. Furthermore, our companion study (Pal et al., 2025, preprint available in BioRivX [cite]) applied PRISM-seq to a novel mouse model engrafted with HIV-infected cells, demonstrating that proviral integration sites play a key determinant role in latency fate decisions in immunocompromised settings, underscoring the biological insight gained through single-template-level mapping of proviral integrations.

PRISM-seq has several limitations. First, clonal expansion estimates currently require replicate reactions. Future iterations incorporating unique molecular identifiers (UMIs) could enable direct quantification of clonal abundance from single reactions. Second, increasing input concentration beyond ≥50 templates per reaction is possible^22^ but means users should turn off the same-gene filter (step 3 in Fig. S9 and see Monte Carlo stimulation Fig S5b) with modest compromise to specificity. Third, while our quality-filtering framework effectively reduces artifacts, it may occasionally exclude rare authentic sites detected by only one proviral end with low read counts - manual check is always recommended. Finally, PRISM-seq provides precise integration sites mapping but does not assess proviral genome intactness, which remains specific to HIV-1 reservoir studies and should be complemented with assays that evaluate proviral sequence integrity ^5,44,45^.

Overall, we have shown that PRISM-seq is an ultra-sensitive, streamlined, and economical method for detecting lentiviral integration sites with single-template resolution. Coupled with its automated bioinformatics pipeline BulkIntSiteR and noise-mitigating steps, the assay delivers a fully integrated workflow for high-confidence integration site identification, filtering, and annotation, especially for rare template detection.

## Method

### Viral-host junction amplification and sequencing

Genomic DNA was extracted (QIAGEN DNeasy kit), quantified for HIV-1 gag by droplet digital PCR (Bio-Rad), subjected to whole-genome amplification using multiple displacement amplification (MDA; QIAGEN REPLI-g Single Cell Kit), and processed using an adaptor-ligation PCR workflow adapted from the Clontech Lenti-X Integration Site Analysis Kit^5^. Final nested PCR was used to selectively enrich viral-host junction products and sequenced on an Illumina MiSeq platform (2 × 150 bp). Full protocol details, origin of PRISM-seq, reagent specifications, and cost are provided in the Extended Methods. Technical replicates were defined as independent MDA reactions from the same DNA extraction; 10 replicates were used for pooling experiments, 2–5 for clinical samples, and none for 8E5/LAV or clones #1–16.

### Identification of HIV-1 IS using BulkIntSiteR

Illumina sequencing-derived FASTQ files were processed using BulkIntSiteR, a one-click R-based, fully automated pipeline designed to detect, map, and annotate HIV-1 integration sites in the human genome. BulkIntSiteR is cross-platform and can handle non-Illumina data. All scripts are available freely on GitHub (https://github.com/guineverelee/BulkIntSiteR/). Briefly, each small read was independently assessed for the presence of high-quality viral ends (containing HXB2 1 or 9719 using BLAST+ suite^46^). For each read positively identified to contain a viral end, the non-viral portion of the read was mapped to the human genome using BLAT^47^. See GitHub documentation for the full algorithm.

### Generation of 14 putative clones with a single integrated provirus

To generate the V1/SBP-GFP HIV-1 reporter virus, 7.5 × 10^7^ 293T cells (ATCC #CRL-3216; RRID: CVCL_0063) were seeded in 875 cm^2^ 5-layer flasks 1 day prior to transfection. 293T cells were transfected with 75 μg pV1/SBP-GFP, 75 μg pCRV1/NLGagPol, and 15 μg pVSV-G (Yee et al., 1994) using polyethyleneimine (PEI). After 12 hours, the medium was replaced with fresh growth medium. Supernatants were collected 48 hours post-transfection, filtered, and concentrated by ultracentrifugation over a 20% sucrose cushion. The pellet containing the virus was resuspended in PBS + 10% BSA, aliquoted, and stored at −80 °C. The viral stock titre (IU/ml) was determined by infecting MT4-LTR-GFP cells and using flow cytometry to assess GFP expression.

For infection, Jurkat and MOLT-4 cells were infected with V1/SBP-GFP at a multiplicity of infection (MOI) of 0.3. Infected cells were enriched using streptavidin bead-based magnetic selection. Single-cell clones of infected Jurkat and MOLT-4 cells were isolated by limiting dilution by diluting to 0.5 cells per well in RPMI growth medium, and 100 μL of the suspension was seeded into each well of 96-well plates. The clonal outgrowth was monitored microscopically, and wells containing single-cell-derived colonies were transferred to fresh wells for expansion.

### Clinical samples

The HIV-1-infected study participant was recruited at the Maple Leaf Clinic in Toronto, Canada. PBMC samples were used according to protocols approved by the respective Institutional Review Boards. CD4^+^ T cells were enriched from total PBMCs using a CD4^+^ T Cell Isolation Kit (STEMCELL Technologies, catalog 17952). Clinical and demographical characteristics of the study participant are summarized in Supplemental Table 3.

## Extended methods (Supplementary)

### Cost and origin

PRISM-seq is an evolution of our previously published matched integration site and proviral sequencing (MIP-seq) protocol^5^, which itself was adapted from the Clontech LentiX integration site analysis method (catalog #631263). MIP-seq was designed to co-capture HIV integration sites together with full-length proviral genomes at single-input-template resolution. In contrast, PRISM-seq is optimized for multiple-template input and focuses specifically on capturing viral integration sites, enabling substantially higher throughput at dramatically reduced cost. At the time of writing, while MIP-seq was prohibitively expensive, with an estimated cost of $240,000 to capture 100 integration sites and their associated proviral genomes. PRISM-seq offers an economical solution at $280 per sample with detection sensitivity down to a single template, making the assay broadly accessible. In addition, PRISM-seq is fully supported by a publicly available bioinformatics (BulkIntSiteR) and quality control pipeline, which automates integration site calling, annotation, and quality assessment.

### Input template quantification by Droplet digital PCR (ddPCR)

DdPCR quantifications were performed to estimate the HIV-1 *gag*-containing proviral template concentration^48^. CD4^+^ T cells were enriched from total PBMCs using a CD4^+^ T Cell Isolation Kit (STEMCELL Technologies, catalog 17952) and subjected to DNA extraction using commercial kits (QIAGEN DNeasy, 69504). We amplified total HIV-1 DNA using ddPCR (Bio-Rad), with primers and probes described previously (127-bp 5′-LTR-gag amplicon; HXB2 coordinates 684–810). PCR was performed using the following program: 95°C for 10 minutes; 45 cycles (95°C for 30 seconds, 60°C for 1 minute); 98°C for 10 minutes. The droplets were subsequently read by a QX100 droplet reader, and data were analyzed using Quanta- Soft software (BIO-RAD).

### Multiple Displacement Amplification

Genomic DNA was isolated from the V1/SBP-GFP infected single cell clone using commercial kits (QIAGEN DNeasy Blood & Tissue Kit, catalog 69504), according to the manufacturer’s instructions. To maximize the likelihood of capturing a single HIV-1 proviral DNA copy, the genomic DNA was amplified using multiple displacement amplification (MDA) with phi29 polymerase (QIAGEN REPLI-g Single Cell Kit, catalog 150345). Buffers DLB, D1 (denaturation buffer), and N1 (neutralization buffer) were prepared according to the manufacturer’s protocol. The genomic DNA (2.5 μL) was denatured by the addition of 2.5 μL of buffer D1, followed by an incubation at room temperature for 3 mins. The mix was neutralized by adding 5 μL of buffer N1. 40 μL of the MDA master mix, consisting of nuclease-free water, REPLI-g single cell Reaction Buffer, and REPLI-g single cell DNA Polymerase, was added to the 10 μL denatured genomic DNA sample. The final 50 μL reaction was incubated at 30°C for 4 h, followed by heat inactivation at 65°C for 3 mins. The amplified product was purified using AMPure XP beads (Beckman Coulter) and was used for downstream integration site analysis.

### Enzymatic digestion of genomic DNA

This protocol was adapted and modified from the Clontech Lenti-X Integration Site Analysis Kit (catalog #631263), which, in its standard form without MDA, lacks sufficient sensitivity to detect single-copy proviral DNA in a sample. Purified MDA products were digested using three blunt-end restriction enzymes - HpaI (NEB #R0105L), SspI (NEB #R3132L), and DraI (NEB #R0129L) - to fragment genomic DNA and facilitate subsequent ligation-based integration site analysis. Briefly, 5 μL of purified MDA product was added to a master mix containing nuclease-free water, 10× restriction enzyme buffer, and either DraI or SspI (20 U μL^−1^) or HpaI (5 U μL^−1^), to a final reaction volume of 50 μL. Reactions were incubated at 37 °C for 18 hours to ensure complete digestion. The digested genomic DNA was then purified using AMPure XP beads and carried forward to adaptor ligation.

### Ligation of the genome-walker adaptor to the digested genomic DNA

The genomic DNA fragments are ligated with double-stranded T-linker DNA (genome walker) (see section below for exact sequence), in which the shorter strand is an oligonucleotide with a 5’ end phosphorylated (to enable efficient linker ligation) and the 3’ end is modified with an amino modification, which limits the extension of the short strand by DNA polymerase and restricts the linker self-ligation amplicons amplification. Digested genomic DNA (4.8 μL) was ligated to adaptors in an 8 μL reaction containing 1.9 μL adaptor (25 μM), 0.8 μL 10× ligation buffer, and 0.5 μL T4 DNA ligase (6 U μL^−1^; NEB #M0202L). Ligation was performed at 16 °C overnight in a thermal cycler to ensure temperature stability. The reaction was terminated by heat inactivation at 70 °C for 5 min, followed by dilution with 32 μL of DEPC-treated water (Ambion #4387937) to a final volume of 40 μL.

### Viral-host junction PCR amplification

The adaptor ligated genomic DNA fragments were used as a template for nested PCR amplification of viral-host junctions. The 5′LTR HIV-1 junction was amplified using LSP1 (GCTTCAGCAAGCCGAGTCCTGCGTCGAG) and LSP2 (GCTCCTCTGGTTTCCCTTTCGCTTTCAA) as forward primers, both derived directly from the CloneTech LentiX kit. The 3′LTR HIV-1 junction was amplified using LestralLTR1 (CTTAAGCCTCAATAAAGCTTGCCTTGAG) and LestralLTR2 (AGACCCTTTTAGTCAGTGTGGAAAATC) ^5^. These were paired with adaptor-specific reverse primers AP1 (GTAATACGACTCACTATAGGGC) and AP2 (ACTATAGGGCACGCGTGGT) for the first and second PCR reactions, respectively. The AP1 primer is specific to the 5’ single-stranded region of the T-linker to avoid unwanted amplification of the host DNA. The adaptor sequence was adapted from the CloneTech LentiX kit (catalog #631263).

Each 25 μL PCR reaction contained 1× reaction buffer, 1× dNTP mix, 0.2 μM of each primer, and Advantage 2 polymerase (Clontech Advantage^®^ 2 PCR Kit, Cat. #639206). For the first PCR, 15 μL of master mix was combined with 10 μL of diluted adaptor-ligated genomic DNA, using AP1/LSP1 for 5′LTR junctions and AP1/LestralLTR1^5^ for 3′LTR junctions. Cycling conditions were: 7 cycles of 94 °C for 25 s and 72 °C for 3 min, followed by 32 cycles of 94 °C for 25 s and 67 °C for 3 min, and a final extension at 67 °C for 7 min.

For the second (nested) PCR, 24 μL of master mix was combined with 1 μL of first-round PCR product, using AP2/LSP2 for 5′LTR junctions and AP2/LestralLTR2^5^ for 3′LTR junctions. Cycling conditions were: 5 cycles of 94 °C for 25 s and 72 °C for 3 min, followed by 20 cycles of 94 °C for 25 s and 67 °C for 3 min, with a final extension at 67 °C for 7 min. Both 5′LTR and 3′LTR viral-host junctions were successfully amplified under these conditions. The resulting PCR products were subjected to Illumina MiSeq paired-end 150 base pairs (bp) sequencing.

### Cell culture

Jurkat cells and MOLT-4 cells were cultured at 37 °C and 5% CO_2_ in RPMI supplemented with 10 % FCS and Gentamycin. All cell lines were monitored by the SG-PERT assay^49^ regularly to ensure the absence of retroviral contamination. Cell lines used in this study were devoid of mycoplasma contamination.

## Supplementary Material

Supplement 1

Supplement 2

## Data Availability

BulkIntSiteR is available under an open-source license at: https://github.com/guineverelee/BulkIntSiteR/
