# Supplementary material for: PRISM-Seq: An Ultra-sensitive Sequencing Approach For Mapping Lentiviral Integration Sites
Source: bioRxiv. 2026 Feb 7:2025.12.20.695659. Originally published 2025 Dec 23. Preprint. [Version 2] doi: 10.64898/2025.12.20.695659 (PMC12776086; doi:10.64898/2025.12.20.695659)
Supplement: 2 [file NIHPP2025.12.20.695659v2-supplement-2.pdf]

bioRxiv preprint doi: https://doi.org/10.64898/2025.12.20.695659; this version posted February 7, 2026. The copyright holder for this preprint (which was not certified by peer review) is the author/funder, who has granted bioRxiv a license to display the preprint in perpetuity. It is made available under aCC-BY 4.0 International license.

## Extended methods (Supplementary)

### Cost and origin

PRISM-seq is an evolution of our previously published matched integration site and proviral sequencing (MIP-seq) protocol[5], which itself was adapted from the Clontech LentiX integration site analysis method (catalog #631263). MIP-seq was designed to co-capture HIV integration sites together with full-length proviral genomes at single-input-template resolution. In contrast, PRISM-seq is optimized for both single and multiple-template input and focuses specifically on capturing viral integration sites, enabling substantially higher throughput at dramatically reduced cost. At the time of writing, PRISM-seq offers an economical solution at $280 per sample with detection sensitivity down to a single template and supports up to ~10000 pre-MDA template input[22], making the assay broadly accessible. In addition, PRISM-seq is fully supported by a publicly available bioinformatics (BulkIntSiteR) and quality control pipeline, which automates integration site calling, annotation, and quality assessment.

### Input template quantification by Droplet digital PCR (ddPCR)

DdPCR quantifications were performed to estimate the HIV-1 *gag* and *env*-containing proviral template concentration[51]. $CD4^+$ T cells were enriched from total PBMCs using a $CD4^+$ T Cell Isolation Kit (STEMCELL Technologies, catalog 17952) and subjected to DNA extraction using commercial kits (QIAGEN DNeasy, 69504). We amplified total HIV-1 DNA using ddPCR (Bio-Rad), with primers and probes previously described (127-bp 5′-LTR-gag amplicon; HXB2 coordinates 684-810[43,52,53] and a modified version of the intact proviral DNA assay (IPDA)[51]we published previously[54]. The droplets were subsequently read by a QX600 droplet reader, and data were analyzed using Quanta-Soft software (BIO-RAD).

bioRxiv preprint doi: https://doi.org/10.64898/2025.12.20.695659; this version posted February 7, 2026. The copyright holder for this preprint (which was not certified by peer review) is the author/funder, who has granted bioRxiv a license to display the preprint in perpetuity. It is made available under aCC-BY 4.0 International license.

**Multiple Displacement Amplification**

Genomic DNA was isolated using commercial kits (QIAGEN DNeasy Blood & Tissue Kit, catalog 69504), according to the manufacturer's instructions. Then, the genomic DNA was amplified using multiple displacement amplification (MDA) with phi29 polymerase (QIAGEN REPLI-g Single Cell Kit, catalog #150345). Buffers DLB, D1 (denaturation buffer), and N1 (neutralization buffer) were prepared according to the manufacturer's protocol. The genomic DNA (2.5 µL) was denatured by the addition of 2.5 µL of buffer D1, followed by an incubation at room temperature for 3 mins. The mix was neutralized by adding 5 µL of buffer N1. 40 µL of the MDA master mix, consisting of nuclease-free water, REPLI-g single cell Reaction Buffer, and REPLI-g single cell DNA Polymerase, was added to the 10 µL denatured genomic DNA sample. The final 50 µL reaction was incubated at 30°C for 4 h, followed by heat inactivation at 65°C for 3 mins. The amplified product was purified using AMPure XP beads (Beckman Coulter) and was used for downstream integration site analysis.

**Enzymatic digestion of genomic DNA**

This protocol was adapted and modified from the Clontech Lenti-X Integration Site Analysis Kit (catalog #631263), which, in its standard form without MDA, lacks sufficient sensitivity to detect single-copy proviral DNA in a sample. Purified MDA products were digested using three blunt-end restriction enzymes - HpaI (NEB #R0105L), SspI (NEB #R3132L), and DraI (NEB #R0129L) - to fragment genomic DNA and facilitate subsequent ligation-based integration site analysis. Briefly, 5 µL of purified MDA product was added to a master mix containing nuclease-free water, 10× restriction enzyme buffer, and either DraI or SspI (20 U $\mu L^{-1}$) or HpaI (5 U $\mu L^{-1}$), to a final reaction volume of 50 µL. Reactions were incubated at 37 °C for 18 hours

bioRxiv preprint doi: https://doi.org/10.64898/2025.12.20.695659; this version posted February 7, 2026. The copyright holder for this preprint (which was not certified by peer review) is the author/funder, who has granted bioRxiv a license to display the preprint in perpetuity. It is made available under aCC-BY 4.0 International license.

to ensure complete digestion. The digested genomic DNA was then purified using AMPure XP beads and carried forward to adaptor ligation.

**Ligation of the genome-walker adaptor to the digested genomic DNA**

The genomic DNA fragments are ligated with double-stranded T-linker DNA (genome walker) (see section below for exact sequence), in which the shorter strand is an oligonucleotide with a 5' end phosphorylated (to enable efficient linker ligation) and the 3' end is modified with an amino modification, which limits the extension of the short strand during PCR in case of linker self-ligation**.** Digested genomic DNA (4.8 µL) was ligated to adaptors in an 8 µL reaction containing 1.9 µL adaptor (25 µM), 0.8 µL 10× ligation buffer, and 0.5 µL T4 DNA ligase (6 U $\mu L^{-1}$; NEB #M0202L). Ligation was performed at 16 °C overnight in a thermal cycler to ensure temperature stability. The reaction was terminated by heat inactivation at 70 °C for 5 min, followed by dilution with 32 µL of DEPC-treated water (Ambion #4387937) to a final volume of 40 µL.

**Viral-host junction PCR amplification**

The adaptor ligated genomic DNA fragments were used as a template for nested PCR amplification of viral-host junctions. The 5′LTR HIV-1 junction was amplified using LSP1 (GCTTCAGCAAGCCGAGTCCTGCGTCGAG) and LSP2 (GCTCCTCTGGTTTCCCTTTCGCTTTCAA) as forward primers, both derived directly from the CloneTech LentiX kit. The 3′LTR HIV-1 junction was amplified using LestralLTR1 (CTTAAGCCTCAATAAAGCTTGCCTTGAG) and LestralLTR2 (AGACCCTTTTAGTCAGTGTGGAAAATC)[5]. These were paired with adaptor-specific reverse primers AP1 (GTAATACGACTCACTATAGGGC) and AP2 (ACTATAGGGCACGCGTGGT) for the first and second PCR reactions, respectively. The

bioRxiv preprint doi: https://doi.org/10.64898/2025.12.20.695659; this version posted February 7, 2026. The copyright holder for this preprint (which was not certified by peer review) is the author/funder, who has granted bioRxiv a license to display the preprint in perpetuity. It is made available under aCC-BY 4.0 International license.

AP1 primer is specific to the 5' single-stranded region of the T-linker to avoid unwanted amplification of the host DNA. The adaptor sequence was adapted from the CloneTech LentiX kit (catalog #631263).

Each 25 µL PCR reaction contained 1× reaction buffer, 1× dNTP mix, 0.2 µM of each primer, and Advantage 2 polymerase (Clontech Advantage® 2 PCR Kit, Cat. #639206). For the first PCR, 15 µL of master mix was combined with 10 µL of diluted adaptor-ligated genomic DNA, using AP1/LSP1 for 5′LTR junctions and AP1/LestralLTR1[5] for 3′LTR junctions. Cycling conditions were: 7 cycles of 94 °C for 25 s and 72 °C for 3 min, followed by 32 cycles of 94 °C for 25 s and 67 °C for 3 min, and a final extension at 67 °C for 7 min.

For the second (nested) PCR, 24 µL of master mix was combined with 1 µL of first-round PCR product, using AP2/LSP2 for 5′LTR junctions and AP2/LestralLTR2[5] for 3′LTR junctions. Cycling conditions were: 5 cycles of 94 °C for 25 s and 72 °C for 3 min, followed by 20 cycles of 94 °C for 25 s and 67 °C for 3 min, with a final extension at 67 °C for 7 min. Both 5′LTR and 3′LTR viral-host junctions were successfully amplified under these conditions. The resulting PCR products were subjected to Illumina MiSeq paired-end 150 base pairs (bp) sequencing.

**Cell culture**

Jurkat cells and MOLT-4 cells were cultured at 37 °C and 5% $CO_2$ in RPMI supplemented with 10 % FCS and Gentamycin. All cell lines were monitored regularly by the SG-PERT assay[55] to ensure the absence of retroviral contamination. Cell lines used in this study were devoid of mycoplasma contamination.

**Availability**

bioRxiv preprint doi: https://doi.org/10.64898/2025.12.20.695659; this version posted February 7, 2026. The copyright holder for this preprint (which was not certified by peer review) is the author/funder, who has granted bioRxiv a license to display the preprint in perpetuity. It is made available under aCC-BY 4.0 International license.

BulkIntSiteR is available under an open-source license at:
https://github.com/guineverelee/BulkIntSiteR/

bioRxiv preprint doi: https://doi.org/10.64898/2025.12.20.695659; this version posted February 7, 2026. The copyright holder for this preprint (which was not certified by peer review) is the author/funder, who has granted bioRxiv a license to display the preprint in perpetuity. It is made available under aCC-BY 4.0 International license.

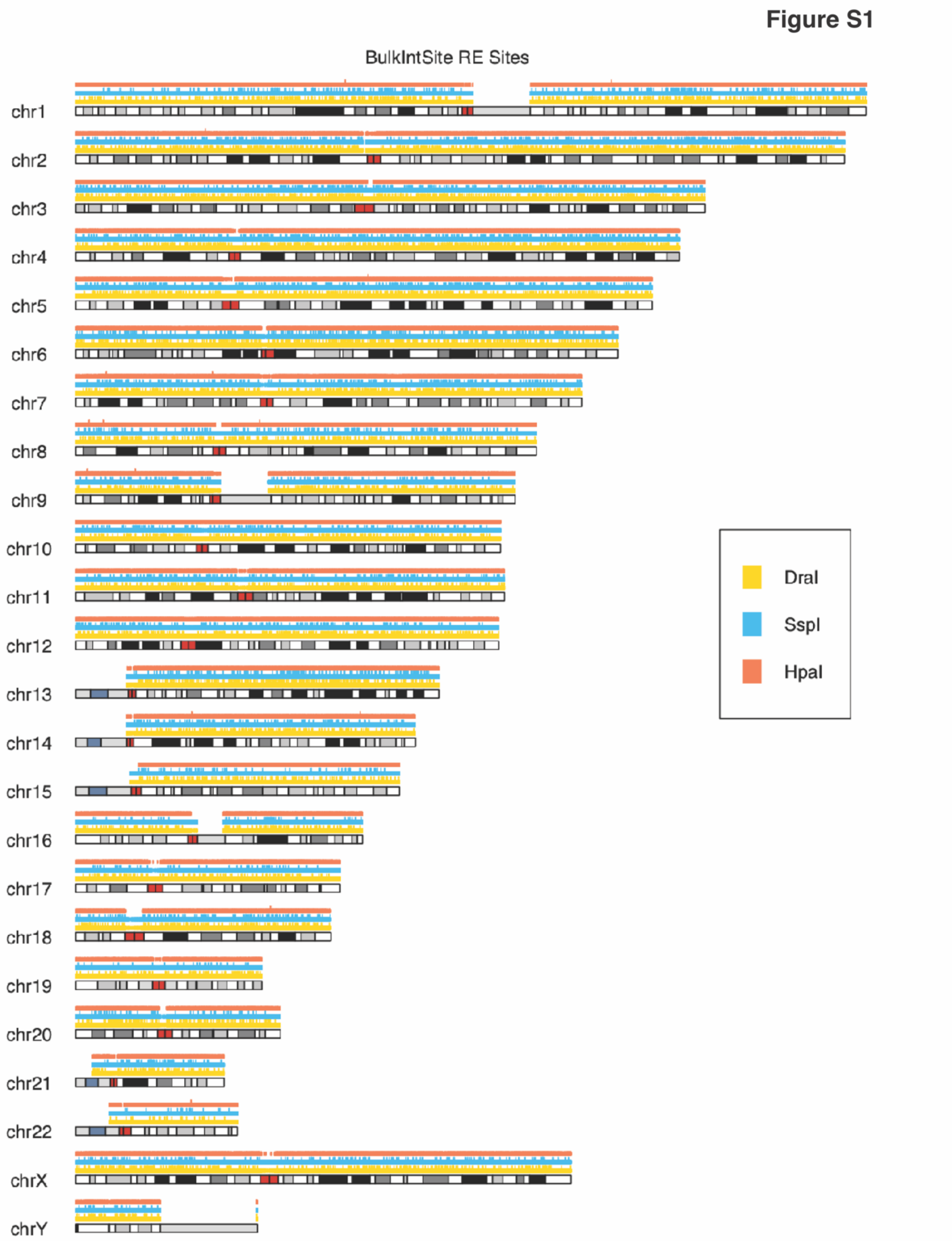


**Figure S1. Genome-wide distribution of the restriction enzyme recognition site.** The karyoplot shows the DraI (yellow), SspI (cyan), and HpaI (orange) restriction sites distribution genome-wide on the human reference genome assembly hg38. *RE, restriction enzymes.*

bioRxiv preprint doi: https://doi.org/10.64898/2025.12.20.695659; this version posted February 7, 2026. The copyright holder for this preprint (which was not certified by peer review) is the author/funder, who has granted bioRxiv a license to display the preprint in perpetuity. It is made available under aCC-BY 4.0 International license.

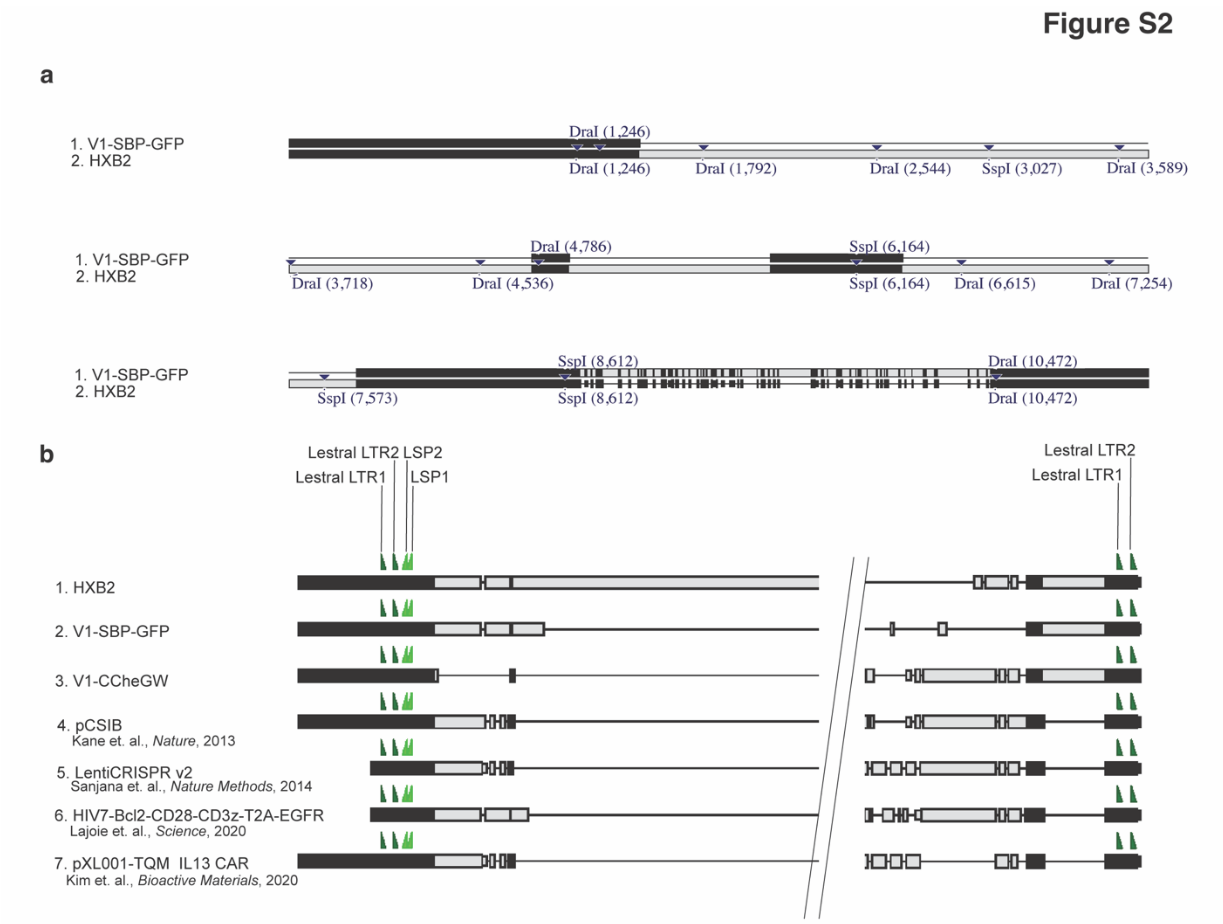


**Figure S2. Distribution of restriction enzyme sites within the provirus genome and PRISM-seq primer binding sites compatibility across HIV and lentiviral vector delivery systems. (a)** Location of DraI and SspI restriction sites in the genome of the full-length HIV-1 reference sequence (HXB2) and replication-deficient HIV-1 reporter (V1-SBP-GFP) from 5′ to 3′ direction. Restriction sites coordinates in the proviral genome are mentioned within brackets next to each restriction enzyme. Black and gray colors represent the identical and non-identical sequences, respectively, between the V1-SBP-GFP and HXB2 genome sequence. **(b)** Multiple sequence alignment of HXB2, replication-deficient single-round HIV-1 reporters (V1- SBP-GFP and V1-CCheGW), pCSIB (lentivirus-based overexpression construct to generate stable cell lines), LentiCRISPRv2 (CRISPR/Cas9-based genome editing lentiviral vector), HIV7-Bcl2-CD28-CD3z-T2A-EGFR, and pXL001-TQM IL13 CAR lentiviral vectors. Primer binding sites for 5′LTR (LSP1 and LSP2) and 3′LTR (LestralLTR1 and LestralLTR2) viral-host junction amplification are conserved across these sequences.

bioRxiv preprint doi: https://doi.org/10.64898/2025.12.20.695659; this version posted February 7, 2026. The copyright holder for this preprint (which was not certified by peer review) is the author/funder, who has granted bioRxiv a license to display the preprint in perpetuity. It is made available under aCC-BY 4.0 International license.

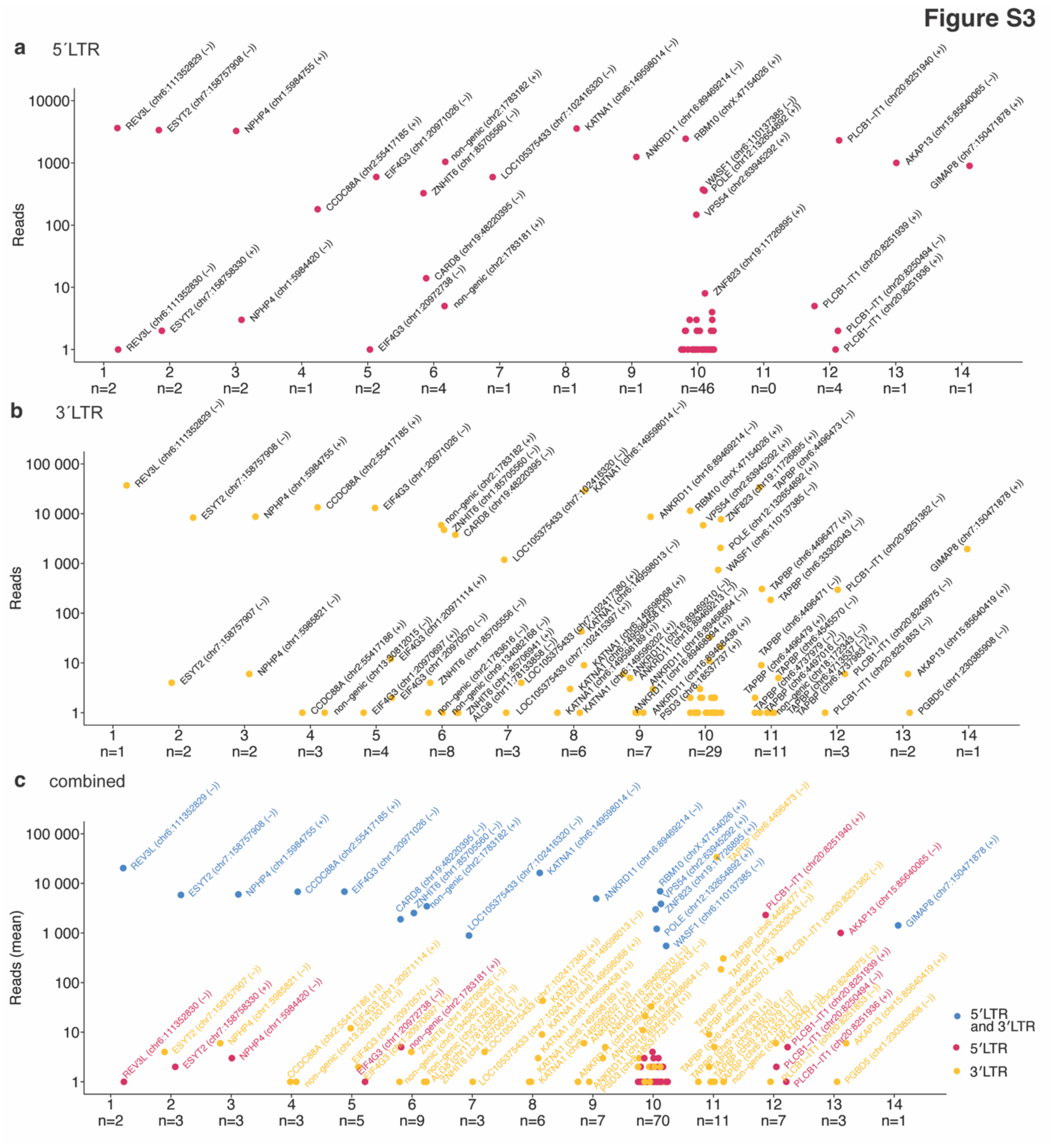


**Figure S3. Identification of proviral integration sites using PRISM-seq in putative single-cell clones #1-14, raw pre-filter data. (a-c)** Proviral integration sites retrieved from individual putative clones, either from the 5′LTR (a) or 3′LTR (b) or combined (c) viral host junction. Blue, red, and yellow dots represent the integration sites identified by both 5′LTR and 3′LTR, exclusively by 5′LTR or 3′LTR, respectively. LTR; long terminal repeat of lentiviruses.

bioRxiv preprint doi: https://doi.org/10.64898/2025.12.20.695659; this version posted February 7, 2026. The copyright holder for this preprint (which was not certified by peer review) is the author/funder, who has granted bioRxiv a license to display the preprint in perpetuity. It is made available under aCC-BY 4.0 International license.

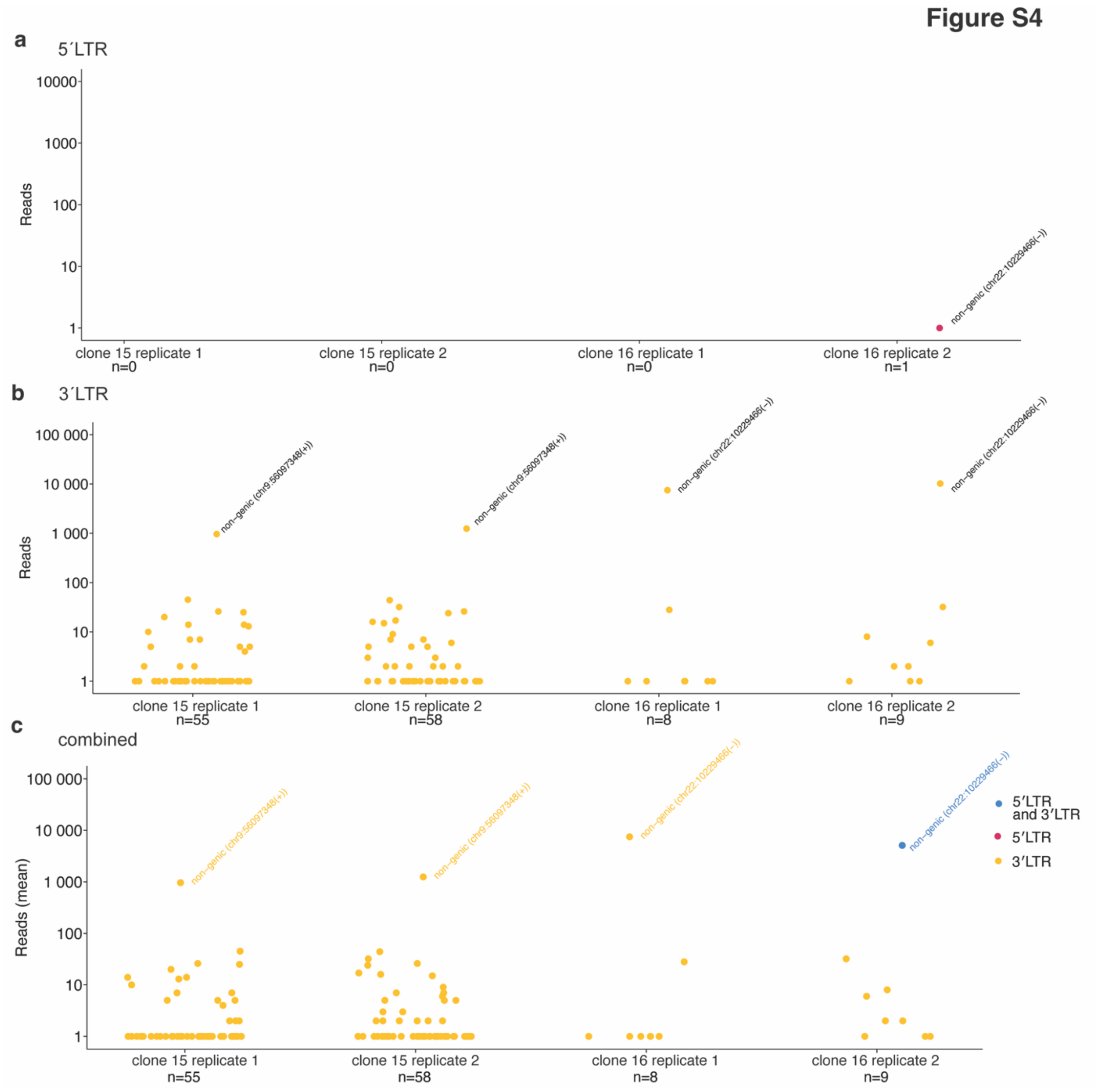


**Figure S4. Identification of proviral integration sites using PRISM-seq in clones with known centromeric integrations #15 and #16, raw pre-filter data**. (a-c) Proviral integration sites were identified in clones #15 and #16, either from the 5′LTR (a) or 3′LTR (b) or combined (c) viral host junction using PRISM-seq. Results for clones #15 and #16 are shown for both technical duplicate reactions.

bioRxiv preprint doi: https://doi.org/10.64898/2025.12.20.695659; this version posted February 7, 2026. The copyright holder for this preprint (which was not certified by peer review) is the author/funder, who has granted bioRxiv a license to display the preprint in perpetuity. It is made available under aCC-BY 4.0 International license.

## Figure S5

**a**

| Types of staggers | examples |
|---|---|
| Integration sites 1 base pair apart (1 bp stagger) | REV3L (chr6:111352829 in negative strand) and REV3L (chr6:111352830 in negative strand) |
| Integration sites 1-10 base pair apart (1-10 bp stagger) | PLCB1-IT1 (chr20:8251940 in positive strand) and PLCB1-IT1 (chr20: 8251936 in positive strand) |
| Integration sites 10-100 bp pair apart (10-100 bp stagger) | EIF4G3 (chr1:20971026 in negative strand and EIF4G3 (chr1:20971114 in positive strand) |
| Integration sites 100- 3000 bp apart (100-3000 bp stagger) | KATNA1(chr6: 149598014 in negative strand and KATNA1 (chr6: 149596202 in positive strand) |

**b**

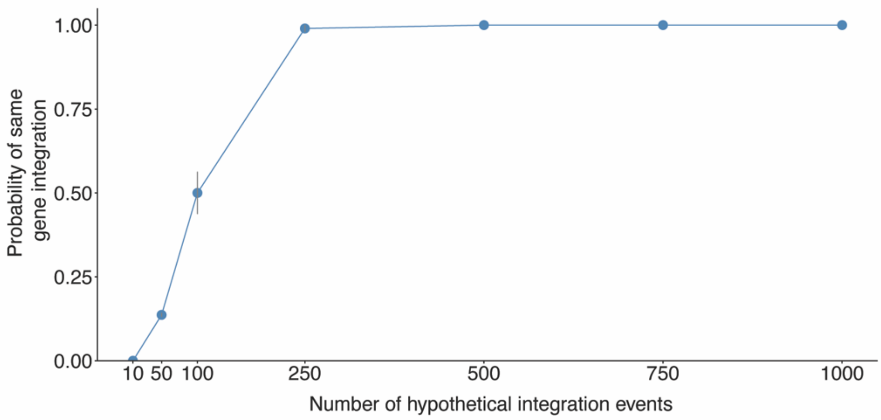


**Figure S5. Identification of look-alike integration site artifact cases and the relationship between integration events and the probability of having true multiple integrations into the same gene. (a)** Characterization of artifacts generated during the integration sites assay. Each staggered case is distinguished based on its genomic distance (bp) from the high-confidence integration sites coordinates of a putative clone. The examples shown here were observed in distinct putative clones. **(b)** Regarding Step 3 in our proposed quality filter, which involves collapsing integration sites that map to the same gene when found within a single reaction: We recognize that when PRISM-seq is applied to non-clonal clinical samples, there is a genuine possibility that proviruses have integrated into distinct locations within the same gene. The likelihood of detecting genuine same gene integration depends on the number of integration events sampled within a PRISM-seq reaction. We performed a Monte Carlo simulation and confirmed that the likelihood of detecting genuine same-gene integration was 0%, 0%, 14%, 50%, and 99% when 1, 10, 50, 100, and 250 unique integration events were sampled. Therefore, we suggest that users should quantify targeting viral DNA copies by techniques such as ddPCR and turn off Step 3 if more than 50 target copies were input into each PRISM-seq reaction, with the caveat that specificity would be compromised. In this study, all reactions were performed at 50 copies per reaction input. *Bp, base pairs.*

bioRxiv preprint doi: https://doi.org/10.64898/2025.12.20.695659; this version posted February 7, 2026. The copyright holder for this preprint (which was not certified by peer review) is the author/funder, who has granted bioRxiv a license to display the preprint in perpetuity. It is made available under aCC-BY 4.0 International license.

# Figure S6

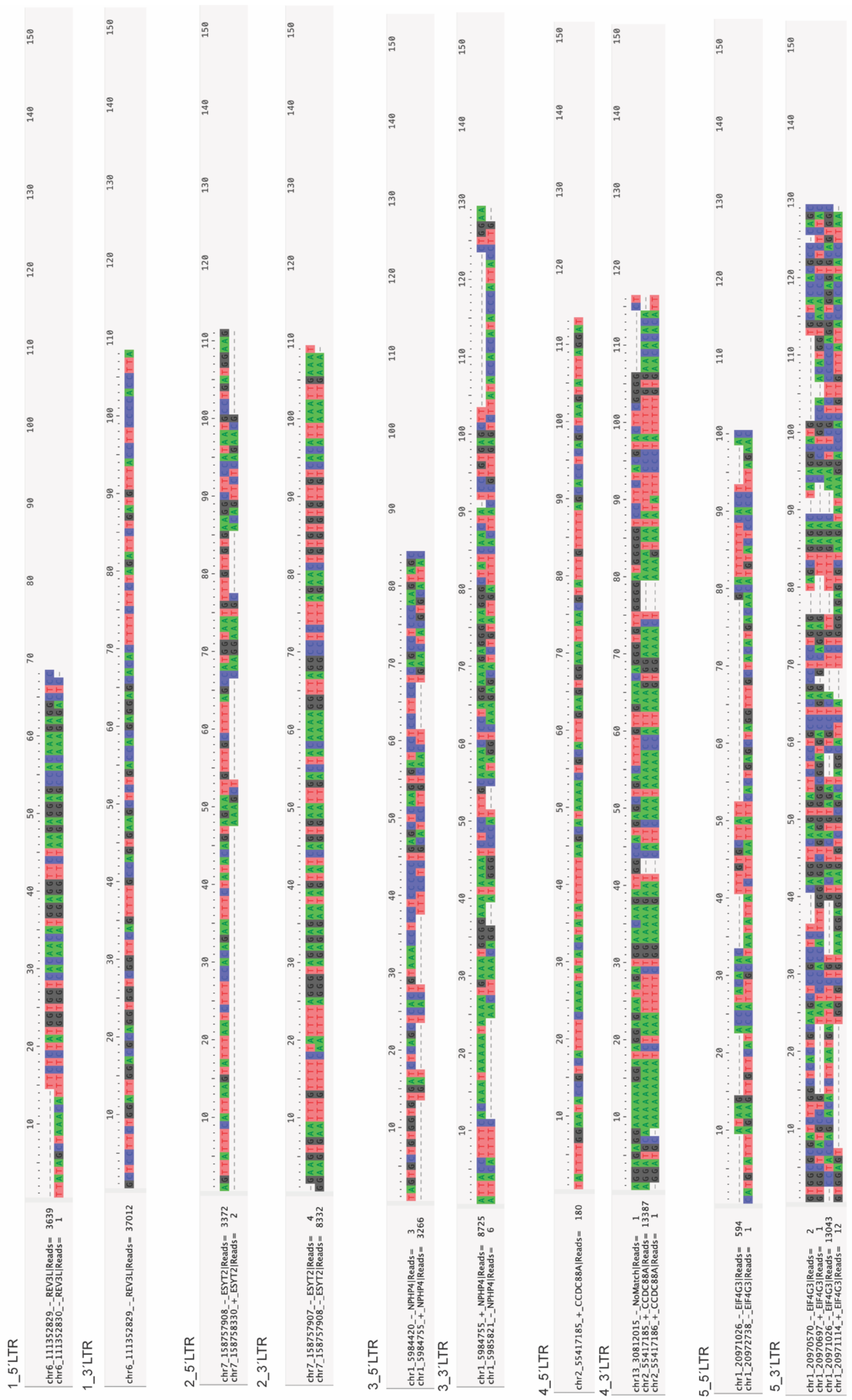

bioRxiv preprint doi: https://doi.org/10.64898/2025.12.20.695659; this version posted February 7, 2026. The copyright holder for this preprint (which was not certified by peer review) is the author/funder, who has granted bioRxiv a license to display the preprint in perpetuity. It is made available under aCC-BY 4.0 International license.

**Figure S6. Comparison of host sequences for each unique integration site identified within the putative clones**. Low-confidence integration sites shared the same junction sequence as the corresponding high-confidence integration site in clones #1, #2, and #4.

bioRxiv preprint doi: https://doi.org/10.64898/2025.12.20.695659; this version posted February 7, 2026. The copyright holder for this preprint (which was not certified by peer review) is the author/funder, who has granted bioRxiv a license to display the preprint in perpetuity. It is made available under aCC-BY 4.0 International license.

**Figure S7**

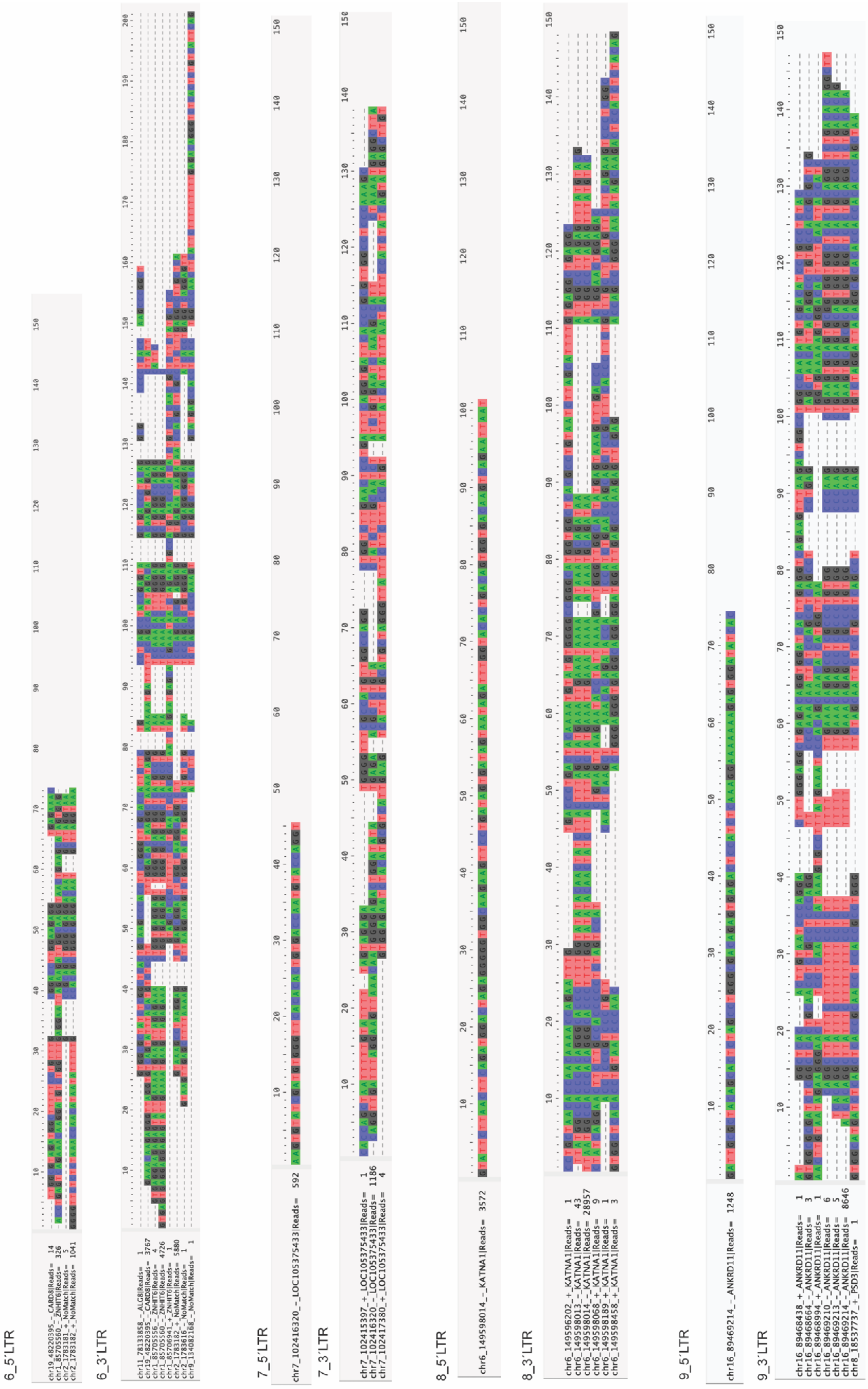

bioRxiv preprint doi: https://doi.org/10.64898/2025.12.20.695659; this version posted February 7, 2026. The copyright holder for this preprint (which was not certified by peer review) is the author/funder, who has granted bioRxiv a license to display the preprint in perpetuity. It is made available under aCC-BY 4.0 International license.

**Figure S7. Comparison of host sequences for each unique integration site identified within the putative clones**. Low-confidence integration sites shared the same junction sequence as the corresponding high-confidence integration site in clones #6, #8, and #9.

bioRxiv preprint doi: https://doi.org/10.64898/2025.12.20.695659; this version posted February 7, 2026. The copyright holder for this preprint (which was not certified by peer review) is the author/funder, who has granted bioRxiv a license to display the preprint in perpetuity. It is made available under aCC-BY 4.0 International license.

**Figure S8**

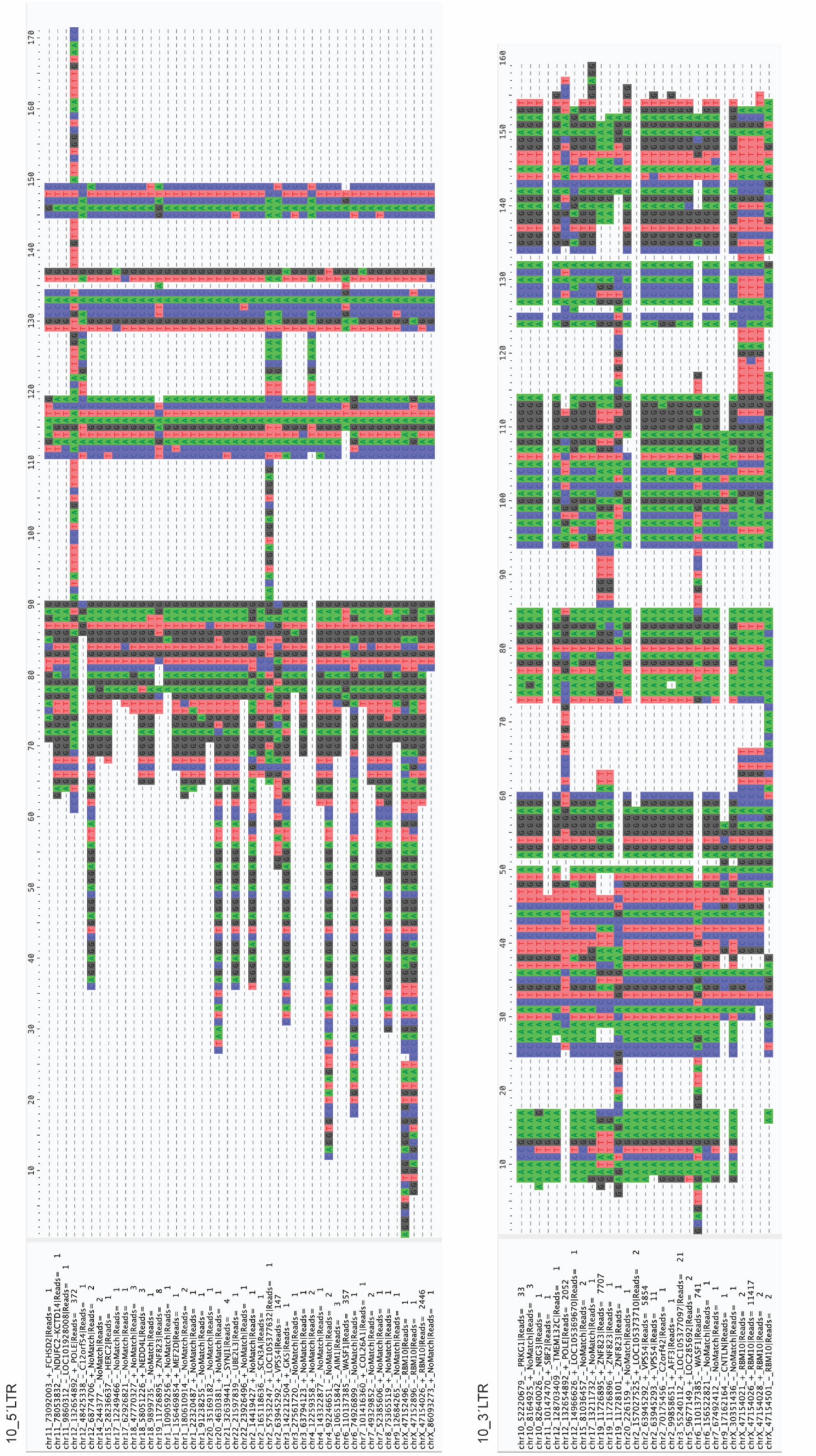

bioRxiv preprint doi: https://doi.org/10.64898/2025.12.20.695659; this version posted February 7, 2026. The copyright holder for this preprint (which was not certified by peer review) is the author/funder, who has granted bioRxiv a license to display the preprint in perpetuity. It is made available under aCC-BY 4.0 International license.

**Figure S8. Comparison of host sequences for each unique integration site identified within the putative clones**. Low-confidence integration sites shared the same junction sequence as the corresponding high-confidence integration site in clone #10.

bioRxiv preprint doi: https://doi.org/10.64898/2025.12.20.695659; this version posted February 7, 2026. The copyright holder for this preprint (which was not certified by peer review) is the author/funder, who has granted bioRxiv a license to display the preprint in perpetuity. It is made available under aCC-BY 4.0 International license.

**Figure S9**

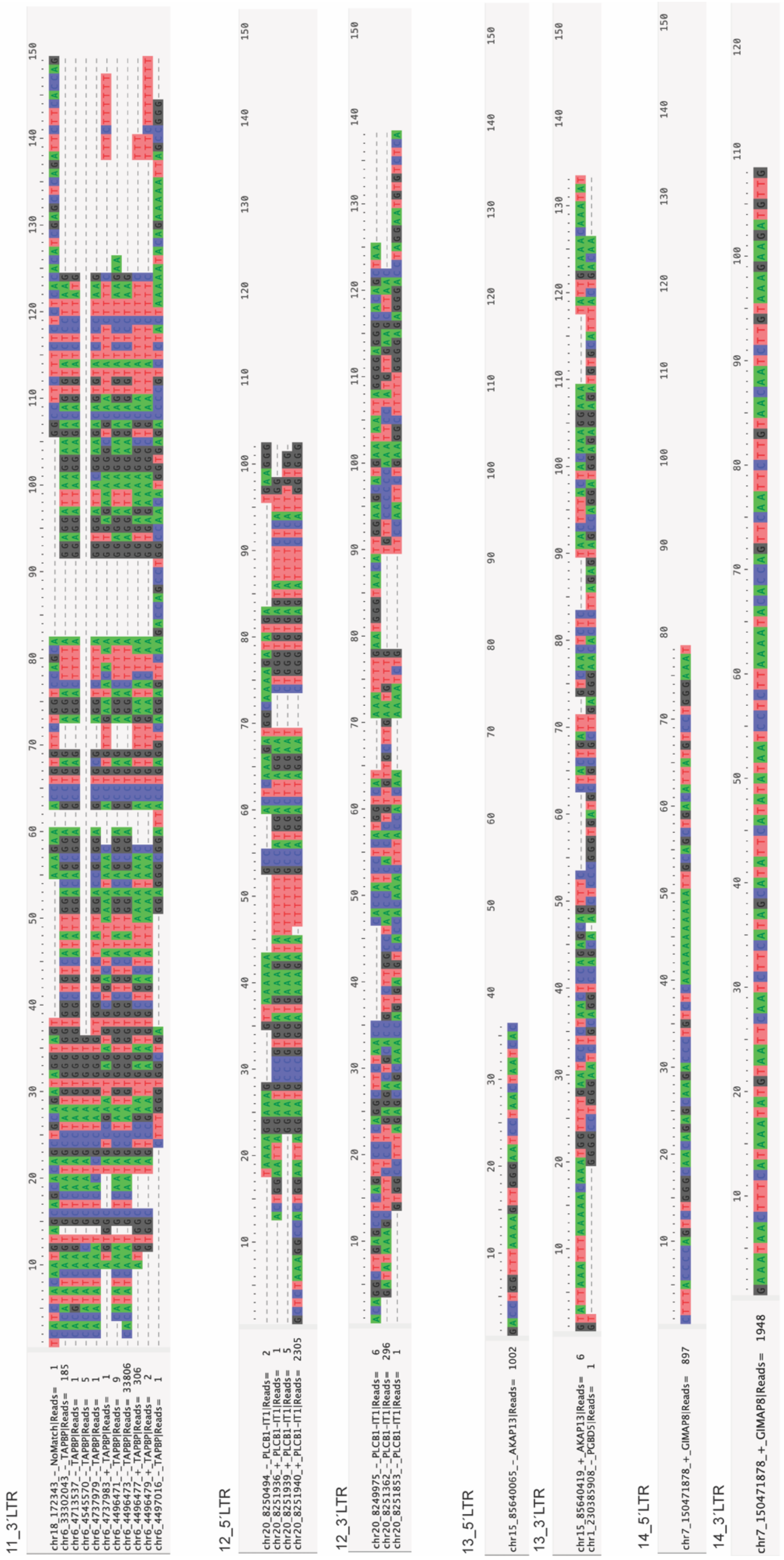

bioRxiv preprint doi: https://doi.org/10.64898/2025.12.20.695659; this version posted February 7, 2026. The copyright holder for this preprint (which was not certified by peer review) is the author/funder, who has granted bioRxiv a license to display the preprint in perpetuity. It is made available under aCC-BY 4.0 International license.

**Figure S9. Comparison of host sequences for each unique integration site identified within the putative clones**. Low-confidence integration sites shared the same junction sequence as the corresponding high-confidence integration site in clones #11 and #12.

bioRxiv preprint doi: https://doi.org/10.64898/2025.12.20.695659; this version posted February 7, 2026. The copyright holder for this preprint (which was not certified by peer review) is the author/funder, who has granted bioRxiv a license to display the preprint in perpetuity. It is made available under aCC-BY 4.0 International license.

**Figure S10**

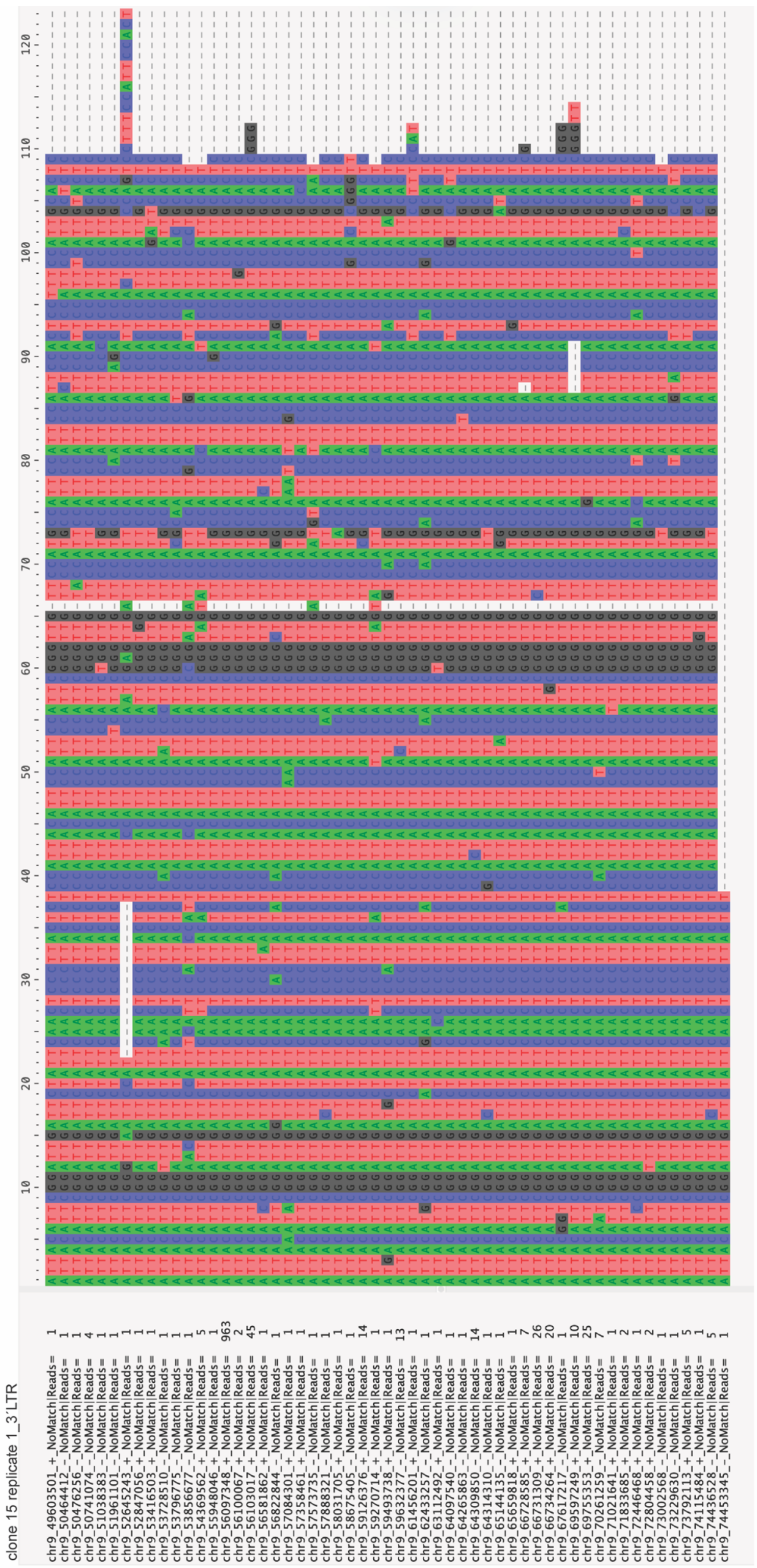

bioRxiv preprint doi: https://doi.org/10.64898/2025.12.20.695659; this version posted February 7, 2026. The copyright holder for this preprint (which was not certified by peer review) is the author/funder, who has granted bioRxiv a license to display the preprint in perpetuity. It is made available under aCC-BY 4.0 International license.

**Figure S10. Comparison of host sequences for each unique integration site identified in a clone associated with centromeric integration.** Low-confidence integration sites shared the same junction sequence as the corresponding high-confidence integration site in clone #15, replicate 1.

bioRxiv preprint doi: https://doi.org/10.64898/2025.12.20.695659; this version posted February 7, 2026. The copyright holder for this preprint (which was not certified by peer review) is the author/funder, who has granted bioRxiv a license to display the preprint in perpetuity. It is made available under aCC-BY 4.0 International license.

**Figure S11**

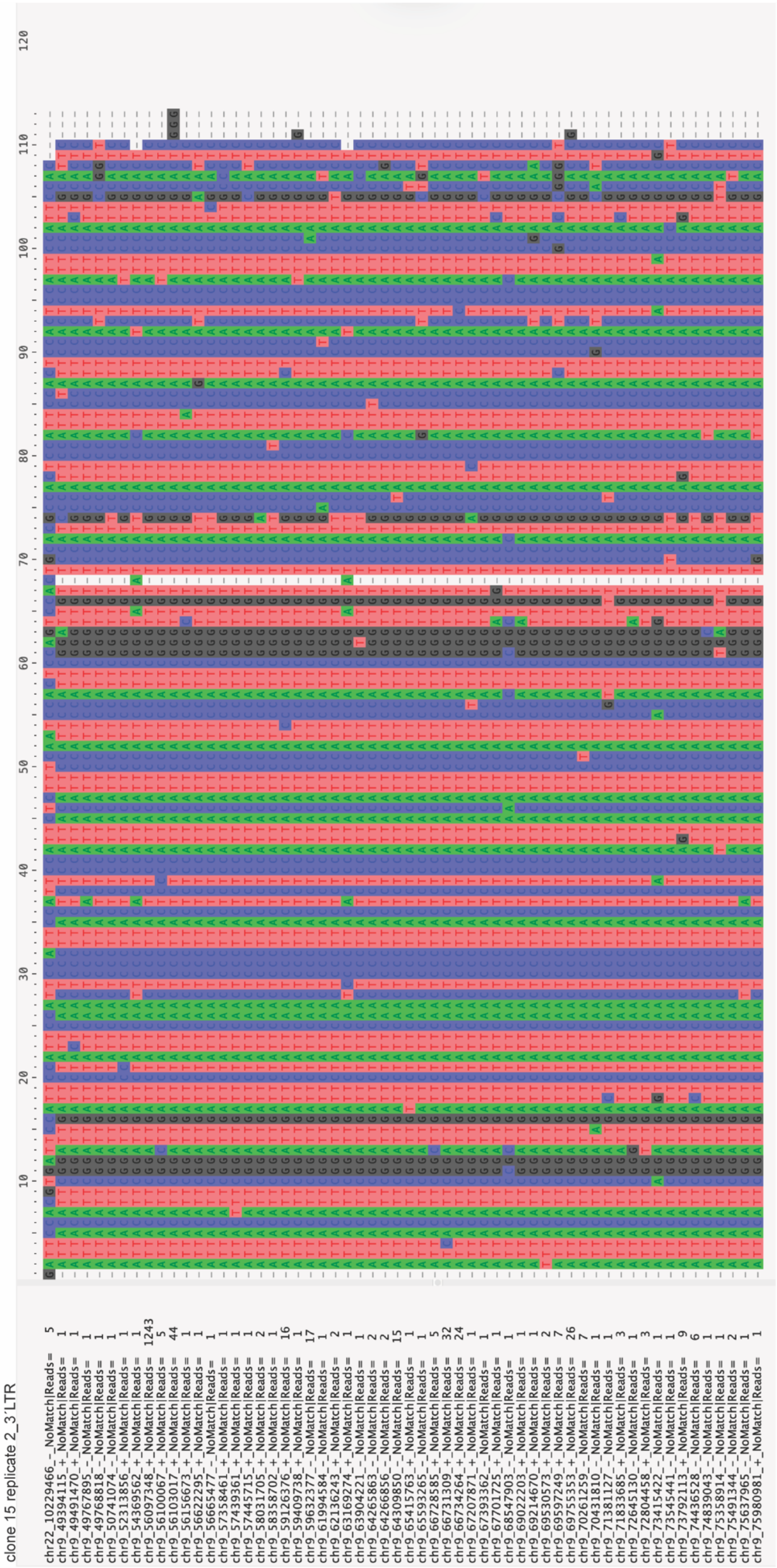

bioRxiv preprint doi: https://doi.org/10.64898/2025.12.20.695659; this version posted February 7, 2026. The copyright holder for this preprint (which was not certified by peer review) is the author/funder, who has granted bioRxiv a license to display the preprint in perpetuity. It is made available under aCC-BY 4.0 International license.

**Figure S11. Comparison of host sequences for each unique integration site identified in a clone associated with centromeric integration.** Low-confidence integration sites shared the same junction sequence as the corresponding high-confidence integration site in clone #15, replicate 2.

bioRxiv preprint doi: https://doi.org/10.64898/2025.12.20.695659; this version posted February 7, 2026. The copyright holder for this preprint (which was not certified by peer review) is the author/funder, who has granted bioRxiv a license to display the preprint in perpetuity. It is made available under aCC-BY 4.0 International license.

**Figure S12**

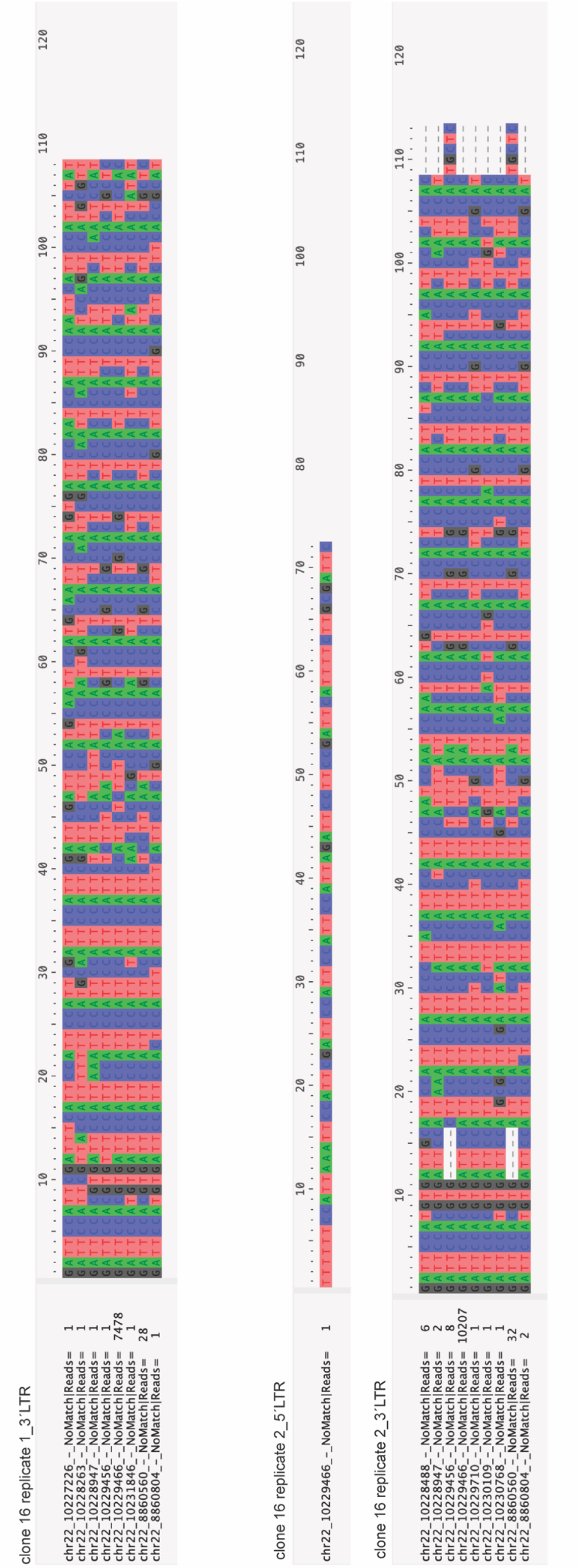

bioRxiv preprint doi: https://doi.org/10.64898/2025.12.20.695659; this version posted February 7, 2026. The copyright holder for this preprint (which was not certified by peer review) is the author/funder, who has granted bioRxiv a license to display the preprint in perpetuity. It is made available under aCC-BY 4.0 International license.

**Figure S12. Comparison of host sequences for each unique integration site identified in a clone associated with centromeric integration.** Low-confidence integration sites shared the same junction sequence as the corresponding high-confidence integration site in clone #16.

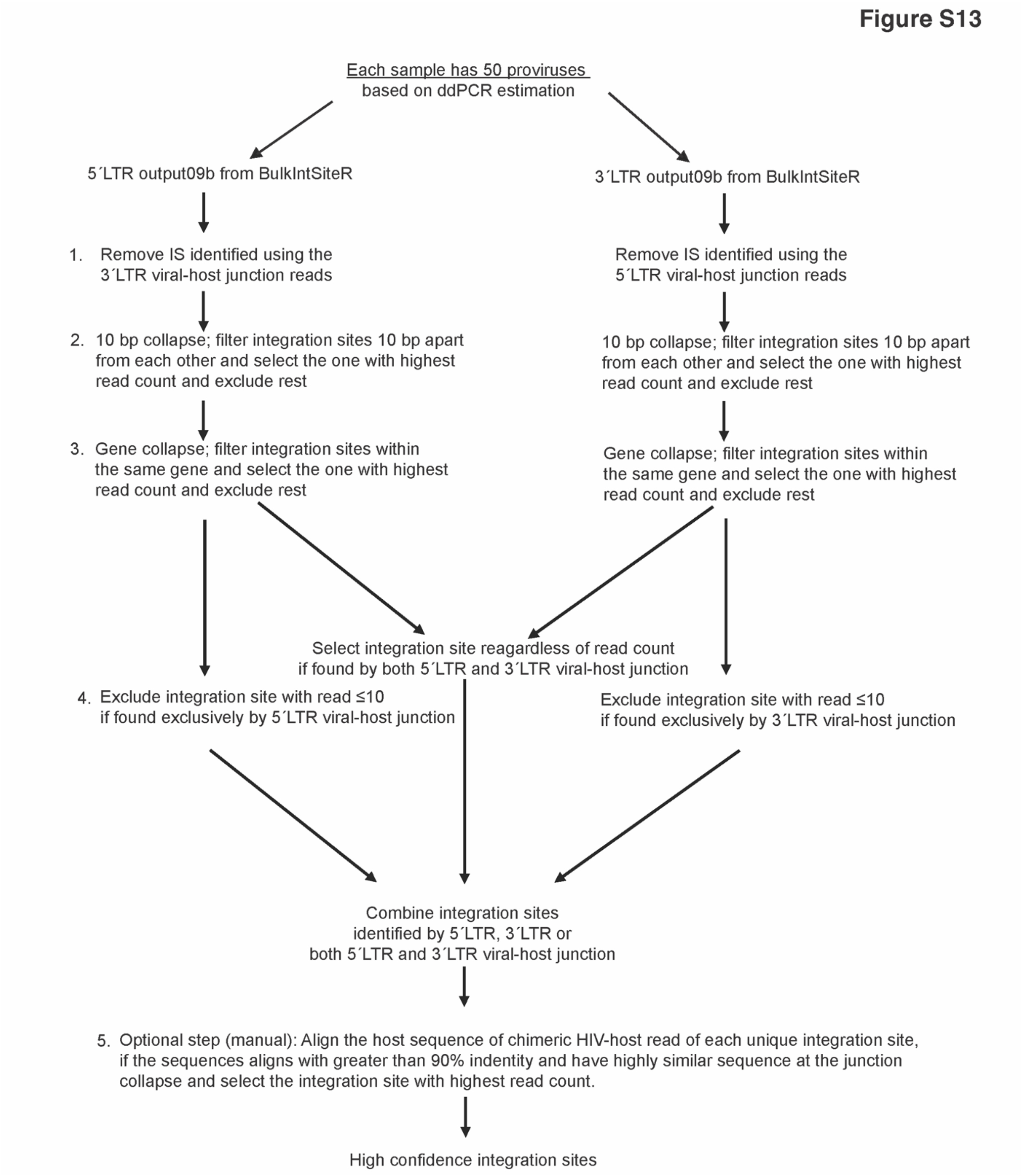


**Figure S13. Data-guided noise removal approach.** Leveraging the clonal nature of Clones #1-16, we devised a quality control filter to remove assay noise. This set of quality filters is applied to the BulkIntSiteR output; (1) filter and remove the spillover noise of integration site identified by 3′ LTR in the 5′ LTR library and vice versa. (2) integration sites within ≤10 bp of

bioRxiv preprint doi: https://doi.org/10.64898/2025.12.20.695659; this version posted February 7, 2026. The copyright holder for this preprint (which was not certified by peer review) is the author/funder, who has granted bioRxiv a license to display the preprint in perpetuity. It is made available under aCC-BY 4.0 International license.

each other were collapsed by retaining the site with the highest read count; (3) integration sites located within the same gene were filtered to retain only one site with the highest read count, excluding all others if the input template count is ≤50 copies; (4) integration sites detected exclusively by either 5′ LTR or 3′ LTR junctions were excluded if supported by ≤10 read counts. Integration sites detected by both 5′ LTR and 3′ LTR virus-host junctions, regardless of the raw read counts, and the sites that passed step 4 were classified as high-confidence. An optional Step (5), involving user-guided homology clustering of host sequences derived from the viral-host chimeric reads, removes look-alike integration sites that share identical junction sequences and co-occur within the same reaction, retaining only the site with the highest read count. Steps 1-4 are fully automated, and the accompanying script is included with the BulkIntSiteR package; Step 5 requires user input and manual curation.

bioRxiv preprint doi: https://doi.org/10.64898/2025.12.20.695659; this version posted February 7, 2026. The copyright holder for this preprint (which was not certified by peer review) is the author/funder, who has granted bioRxiv a license to display the preprint in perpetuity. It is made available under aCC-BY 4.0 International license.

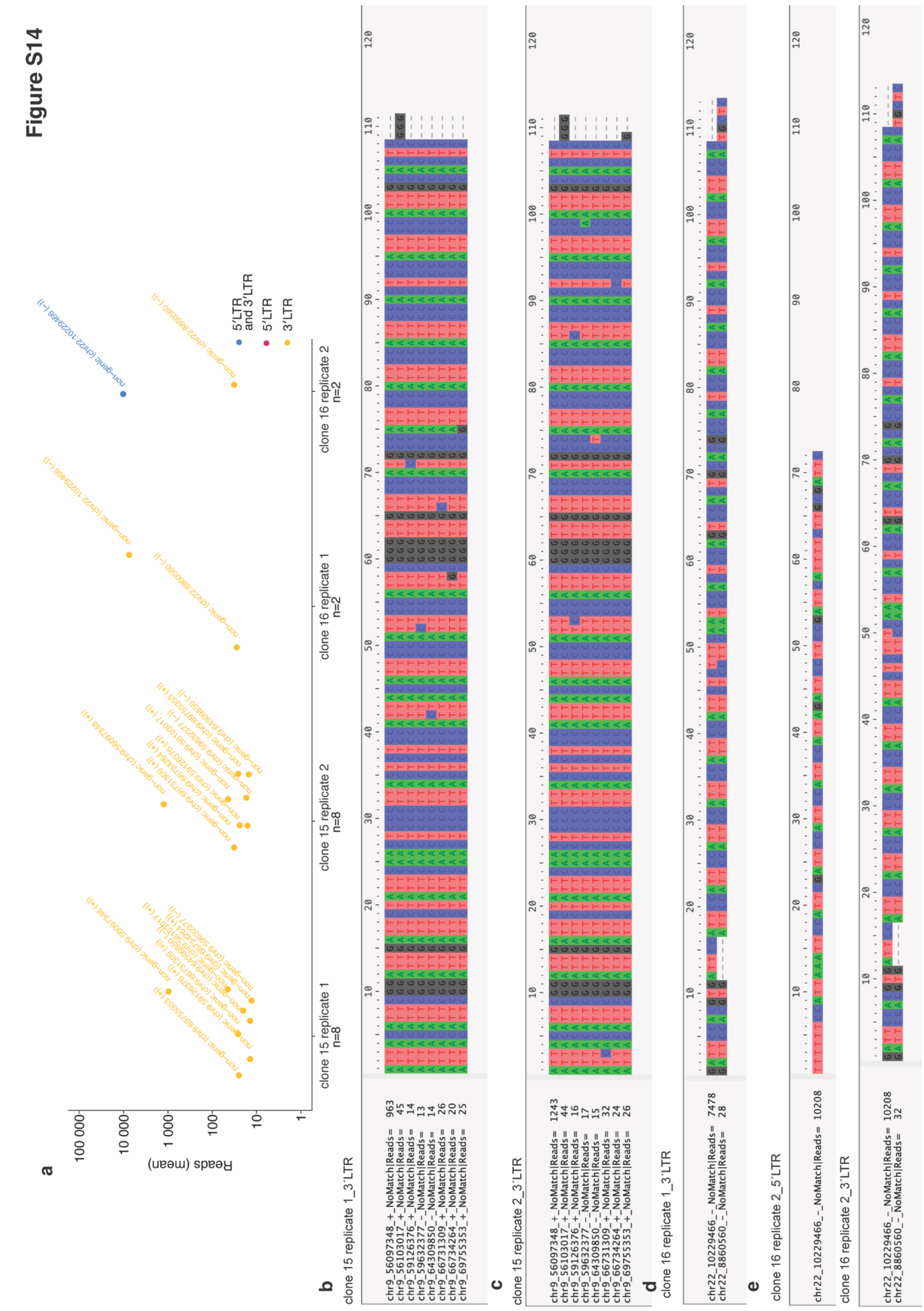

bioRxiv preprint doi: https://doi.org/10.64898/2025.12.20.695659; this version posted February 7, 2026. The copyright holder for this preprint (which was not certified by peer review) is the author/funder, who has granted bioRxiv a license to display the preprint in perpetuity. It is made available under aCC-BY 4.0 International license.

**Figure S14. Post-filter recovery of proviral integration sites found within centromeric clones #15 and #16. (a)** Integration sites identified by the BulkIntSiteR software were processed using the QC filtering pipeline (with steps 1-4 applied), and results for clones #15 and #16 are shown for technical duplicate reactions. **(b-e)** Comparison of the host sequence for all unique integration sites after QC filtering for clones #15 and #16. The unexpected low-confidence integration sites share the same junction sequence as the high-confidence integration site for clones #15 and #16.

bioRxiv preprint doi: https://doi.org/10.64898/2025.12.20.695659; this version posted February 7, 2026. The copyright holder for this preprint (which was not certified by peer review) is the author/funder, who has granted bioRxiv a license to display the preprint in perpetuity. It is made available under aCC-BY 4.0 International license.

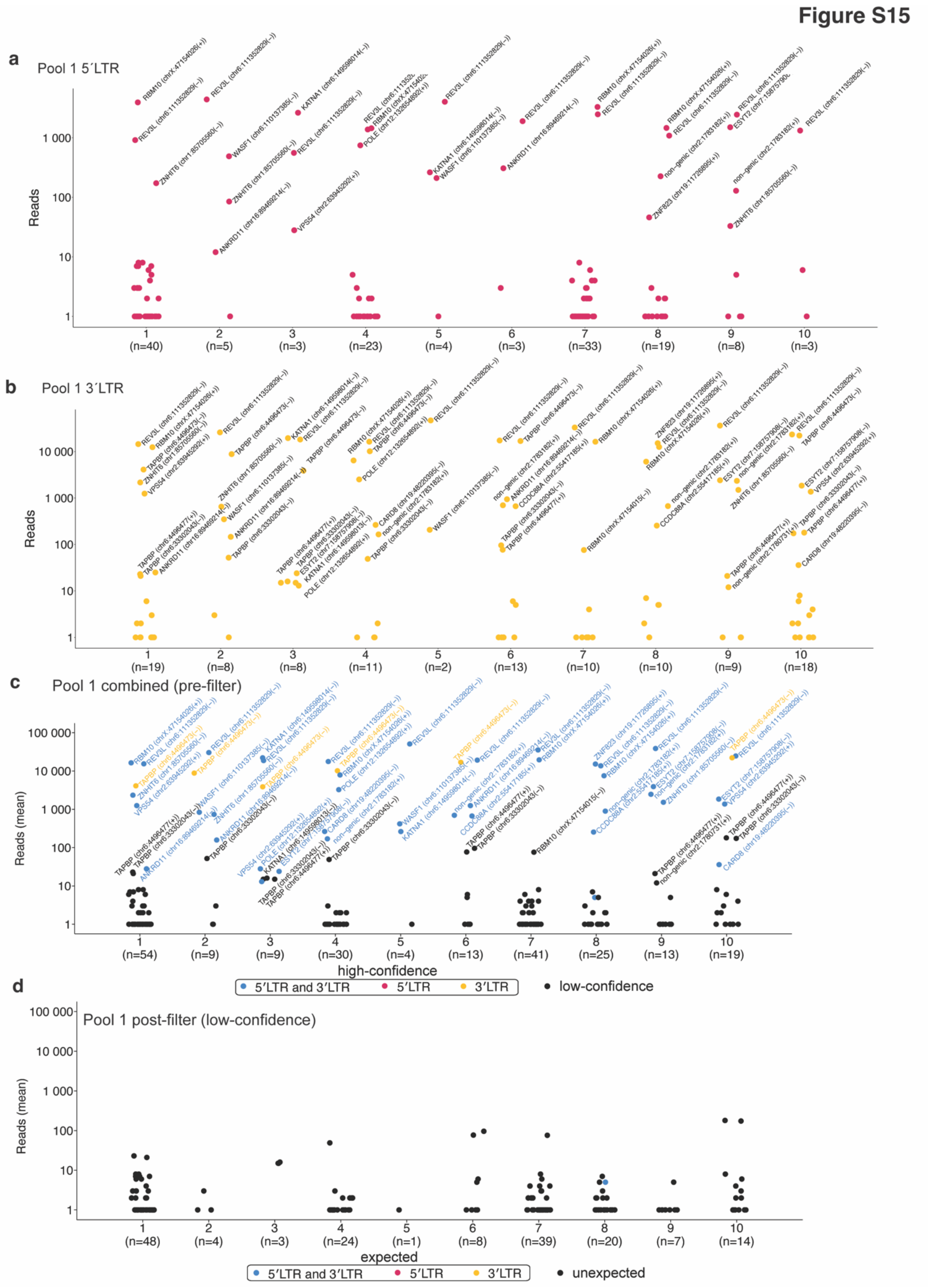


909

910 **Figure S15. Proviral integration sites identification in pool 1. (a-c)** Dot plots representing
911 the total number of unique integration sites identified in each replicate of pool 1 by either the
912 5′LTR (a) or 3′LTR (b) or combined (c) viral-host junction. The total number of unique

bioRxiv preprint doi: https://doi.org/10.64898/2025.12.20.695659; this version posted February 7, 2026. The copyright holder for this preprint (which was not certified by peer review) is the author/funder, who has granted bioRxiv a license to display the preprint in perpetuity. It is made available under aCC-BY 4.0 International license.

integration sites retrieved in an individual replicate is represented as an n value. Blue, red, and yellow dots represent integration sites identified by both 5′LTR and 3′LTR, exclusively by 5′LTR or exclusively by 3′LTR PRISM-seq reactions, respectively. (d) Post-filter identification of low-confidence expected and unexpected integration sites.

bioRxiv preprint doi: https://doi.org/10.64898/2025.12.20.695659; this version posted February 7, 2026. The copyright holder for this preprint (which was not certified by peer review) is the author/funder, who has granted bioRxiv a license to display the preprint in perpetuity. It is made available under aCC-BY 4.0 International license.

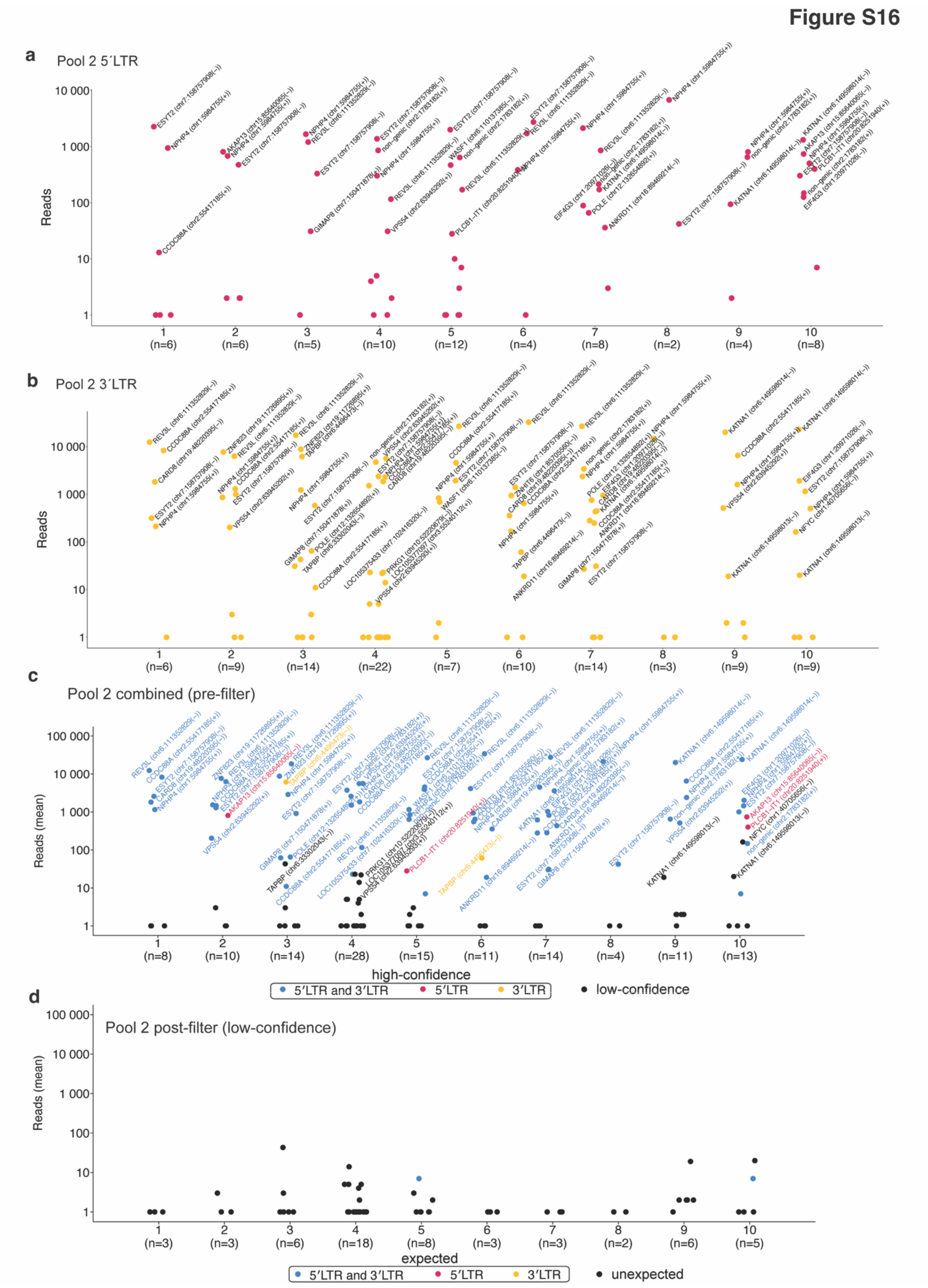


**Figure S16. Proviral integration sites identification in pool 2. (a-c)** Dot plots representing the total number of unique integration sites identified in each replicate of pool 2 by either the 5′LTR (a) or 3′LTR (b) or combined (c) viral-host junction. The total number of unique

bioRxiv preprint doi: https://doi.org/10.64898/2025.12.20.695659; this version posted February 7, 2026. The copyright holder for this preprint (which was not certified by peer review) is the author/funder, who has granted bioRxiv a license to display the preprint in perpetuity. It is made available under aCC-BY 4.0 International license.

integration sites retrieved in an individual replicate is represented as an n value. Blue, red, and yellow dots represent the integration sites identified by both 5′LTR and 3′LTR, exclusively by 5′LTR or exclusively by 3′LTR PRISM-seq reactions, respectively. (d) Post-filter identification of low-confidence expected and unexpected integration sites.

bioRxiv preprint doi: https://doi.org/10.64898/2025.12.20.695659; this version posted February 7, 2026. The copyright holder for this preprint (which was not certified by peer review) is the author/funder, who has granted bioRxiv a license to display the preprint in perpetuity. It is made available under aCC-BY 4.0 International license.

**Figure S17**

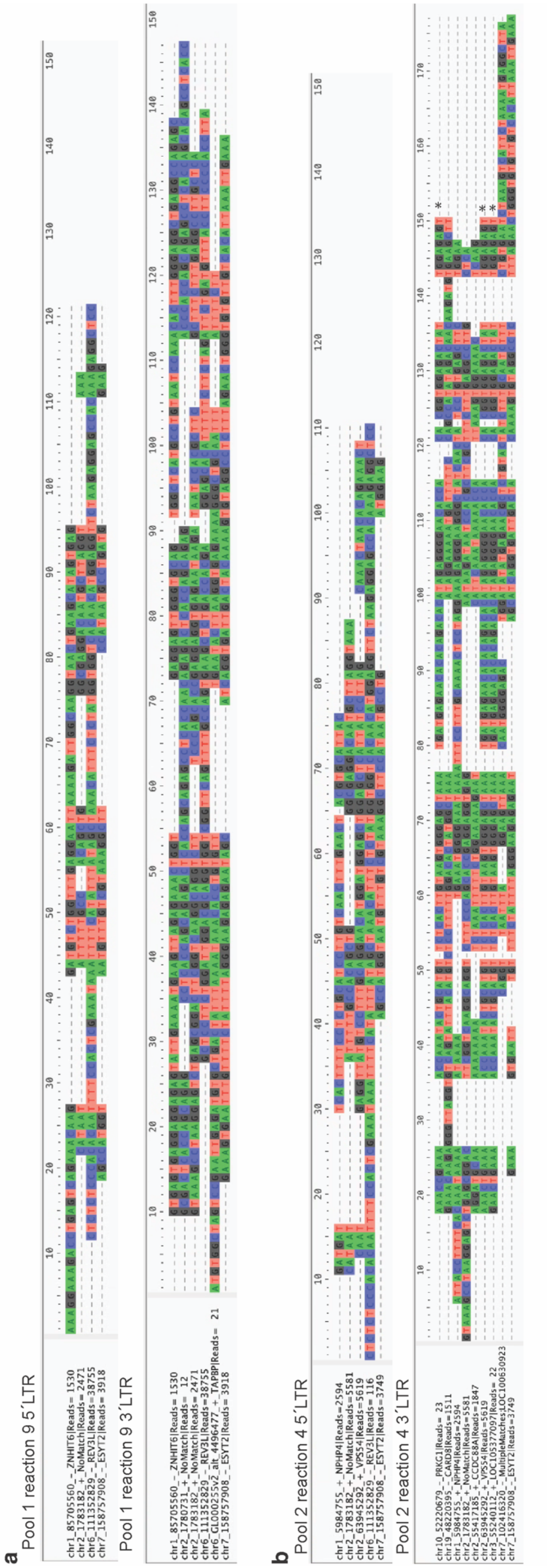

bioRxiv preprint doi: https://doi.org/10.64898/2025.12.20.695659; this version posted February 7, 2026. The copyright holder for this preprint (which was not certified by peer review) is the author/funder, who has granted bioRxiv a license to display the preprint in perpetuity. It is made available under aCC-BY 4.0 International license.

**Figure S17. Comparison of host sequences of unexpected integration sites observed in pool 1 and pool 2, respectively (a and b).** Host sequence derived from unexpected integration sites found in pool 1 replicate 9 (a) and pool 2 replicate 4 (b) by both 5′LTR and 3′LTR PRISM-seq reactions. Unexpected integration sites sharing the same junction sequence as the high-confidence site and/or are ±10 kb away from the expected high-confidence integration site are labelled with an asterisk (*).
