# Supplementary material for: PRISM-Seq: An Ultra-sensitive Sequencing Approach For Mapping Lentiviral Integration Sites
Source: bioRxiv. 2025 Dec 23:2025.12.20.695659. Preprint. [Version 1] doi: 10.64898/2025.12.20.695659 (PMC12776086; doi:10.64898/2025.12.20.695659)
Supplement: Supplementary file 1 [file NIHPP2025.12.20.695659v1-supplement-2.pdf]

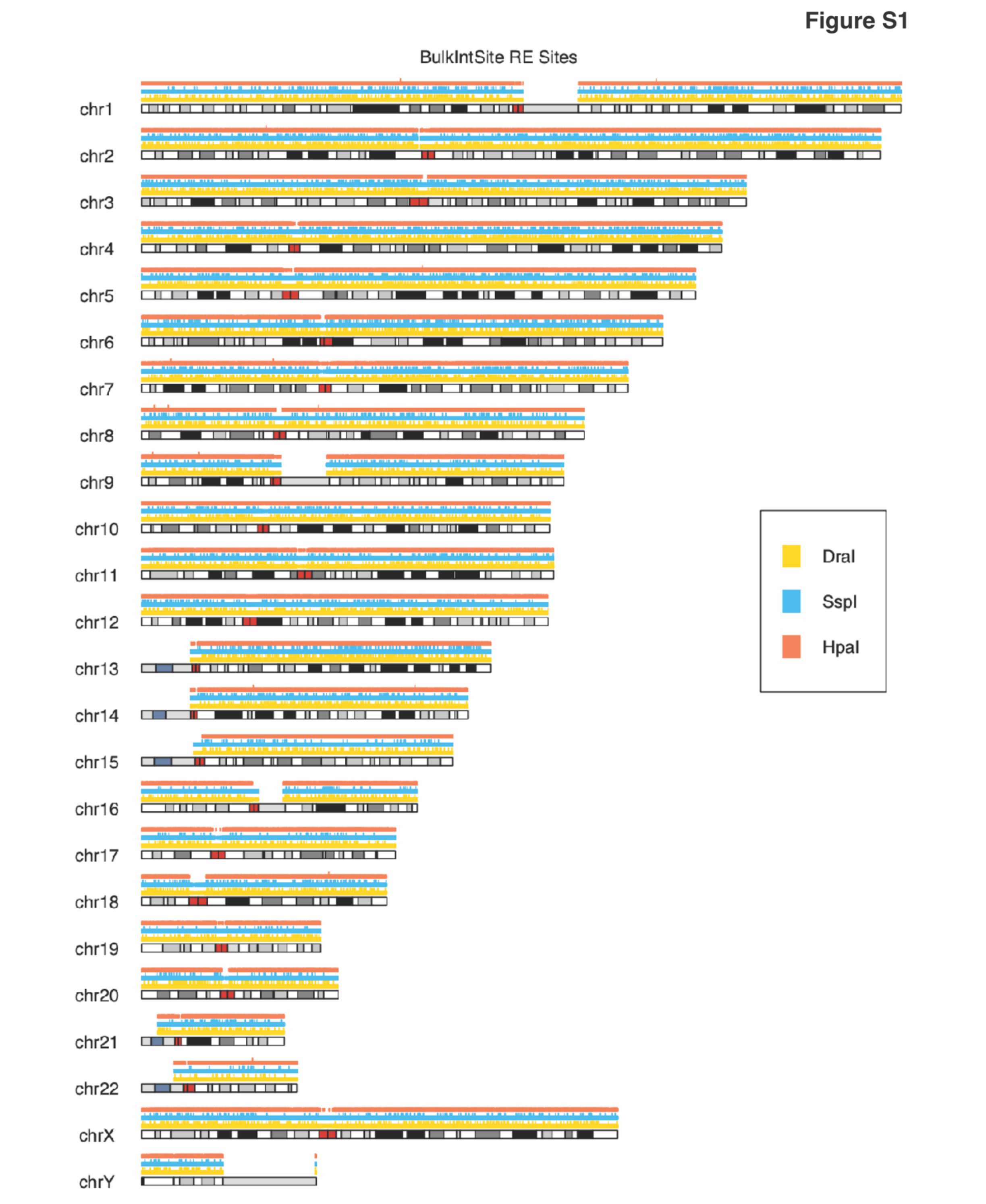

Figure S1. Genome-wide distribution of the restriction enzyme recognition site. The karyoplot shows the DraI (yellow), SspI (cyan), and HpaI (orange) restriction sites distribution genome-wide on the human reference genome assembly hg38. RE, restriction enzymes.

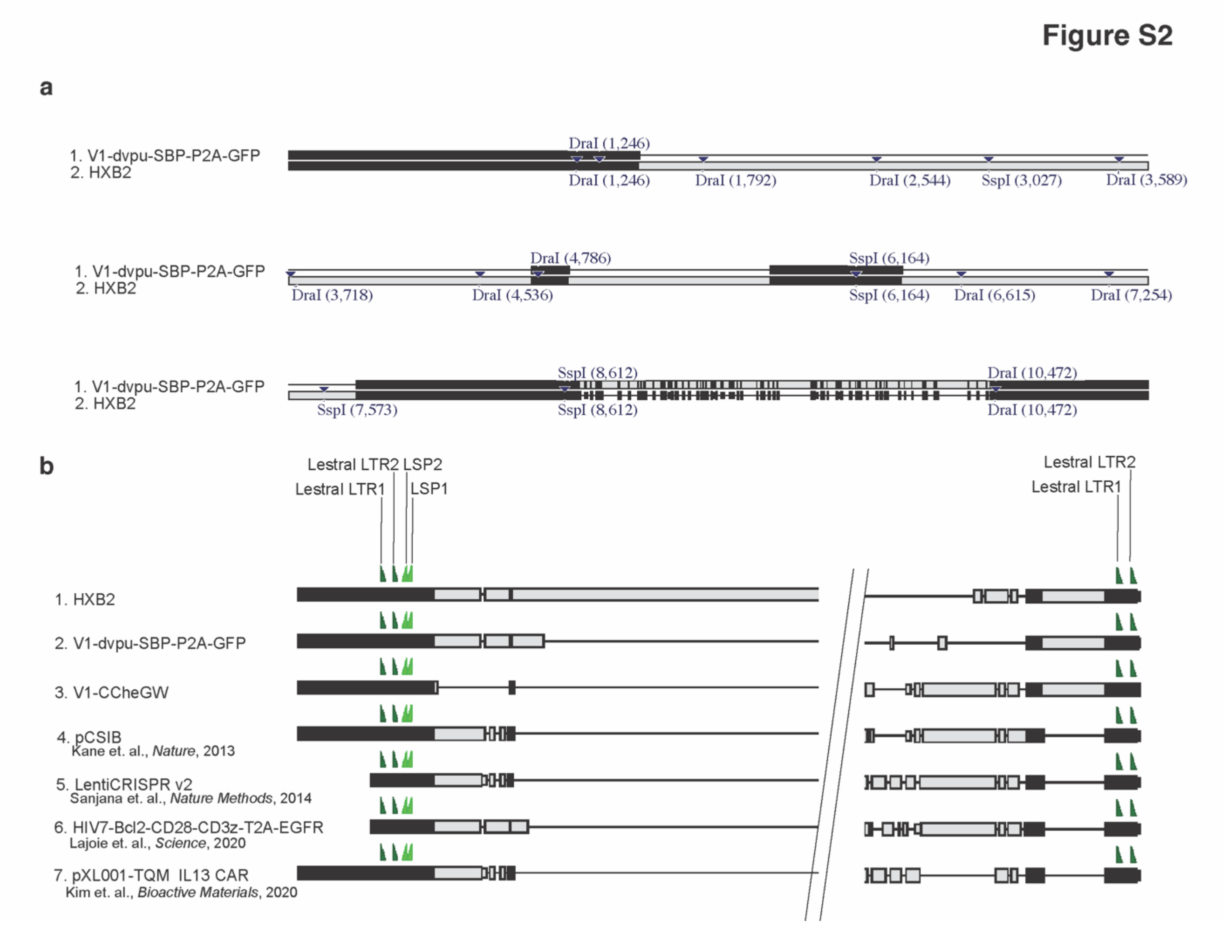

Figure S2. Distribution of restriction enzyme sites within the provirus genome and PRISM-seq primer binding sites compatibility across HIV and lentiviral vector delivery systems. (a) Location of DraI and SspI restriction sites in the genome of the full-length HIV-1 reference sequence (HXB2) and replication-deficient HIV-1 reporter (V1-SBP-GFP) from 5′ to 3′ direction. The restriction sites coordinate in the proviral genome are mentioned within brackets next to each restriction enzyme. The black and gray colors represent the identical and non-identical sequences, respectively, between the V1-SBP-GFP and HXB2 genome sequence. **(b)** Multiple sequence alignment of HXB2, replication-deficient single-round HIV-1 reporters (V1- SBP-GFP and V1-CCheGW), pCSIB (lentivirus-based overexpression construct to generate stable cell lines), LentiCRISPRv2 (CRISPR/Cas9-based genome editing lentiviral vector), HIV7-Bcl2-CD28-CD3z-T2A-EGFR, and pXL001-TQM IL13 CAR lentiviral vectors. The primer binding sites for 5′LTR (LSP1 and LSP2) and 3′LTR (LestralLTR1 and LestralLTR2) viral-host junction amplification are conserved across these sequences.

Figure S3

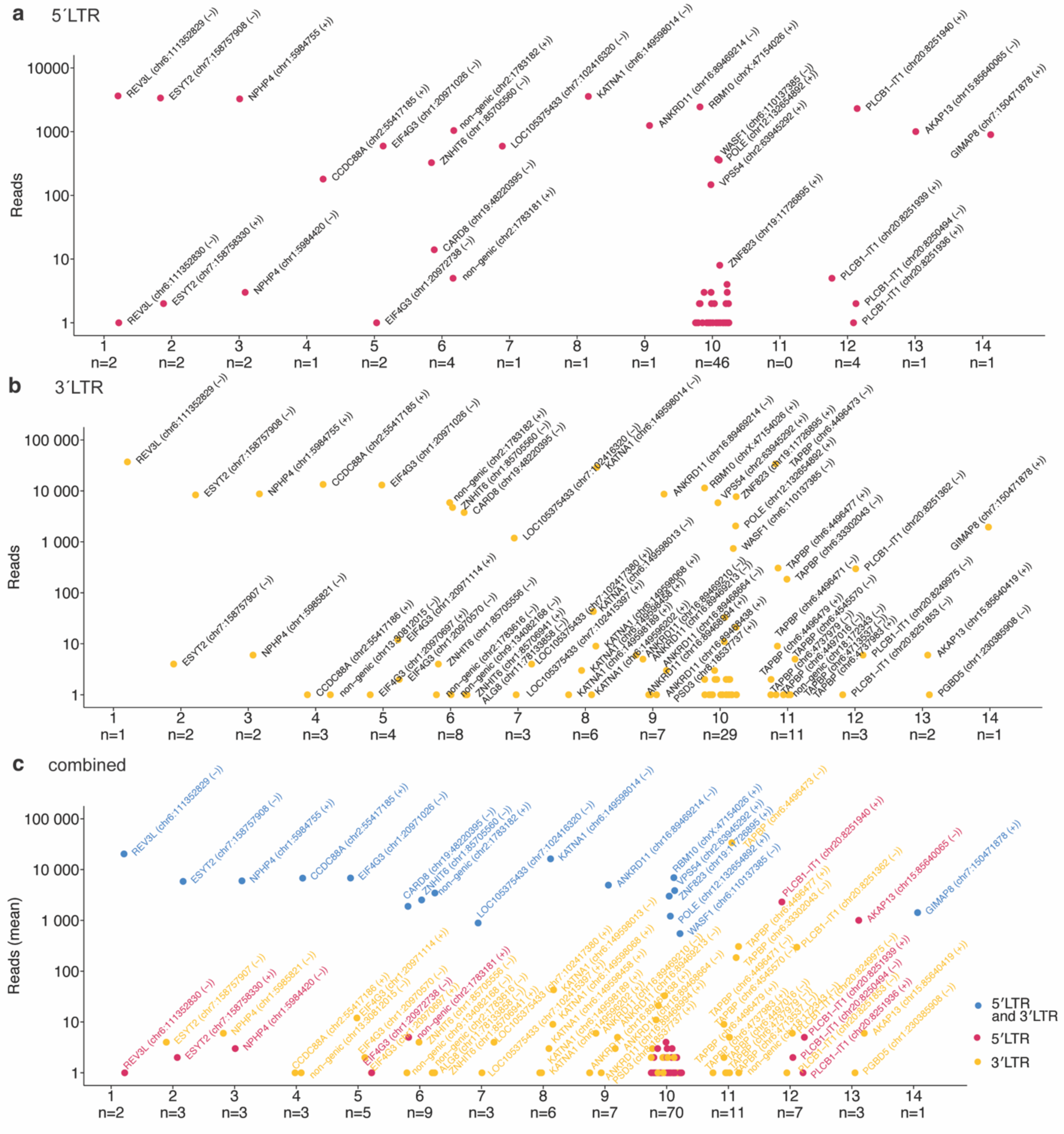

Figure S3. Identification of proviral integration sites using PRISM-seq in putative single-cell clones #1-14, raw data. (a-c) The proviral integration sites retrieved from individual putative clones, either from the 5′LTR (a) or 3′LTR (b) or combined (c) viral host junction. The blue, red, and yellow dots represent the integration sites identified by both 5′LTR and 3′LTR, exclusively by 5′LTR or 3′LTR, respectively. LTR; long terminal repeat of lentiviruses.

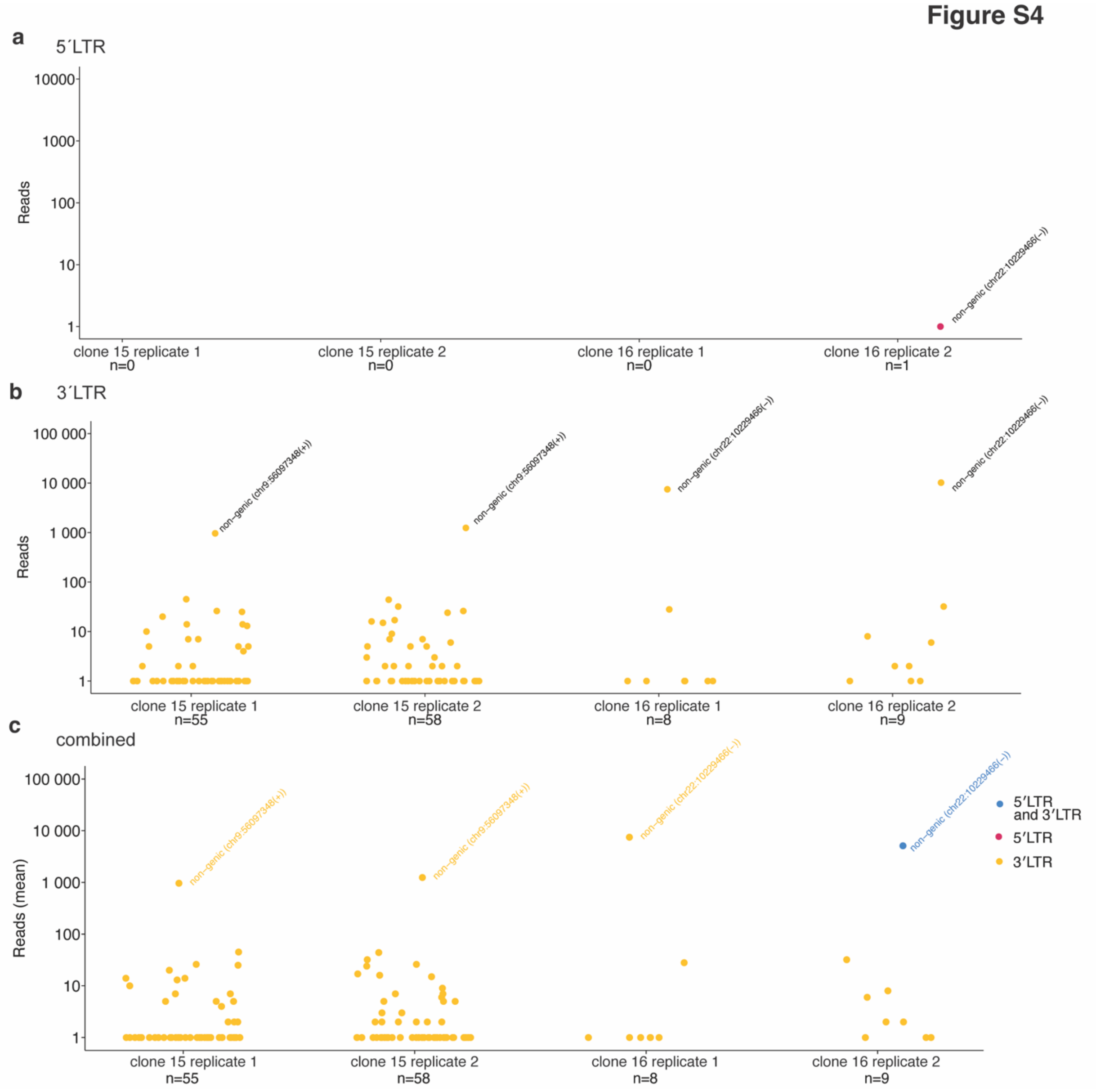

Figure S4. Identification of proviral integration sites using PRISM-seq in clones with known centromeric integrations #15 and #16, raw data. (a-c) The proviral integration sites were identified in clones #15 and #16, either from the 5′LTR (a) or 3′LTR (b) or combined (c) viral host junction using PRISM-seq. Results for clones #15 and #16 are shown for both technical duplicate reactions.

Figure S5

a

Number of cases	Types of staggers	examples
1	Integration sites 1 base pair apart (1 bp stagger)	REV3L (chr6:111352829 in negative strand) and REV3L (chr6:111352830 in negative strand)
2	Integration sites 1-10 base pair apart (1-10 bp stagger)	PLCB1-IT1 (chr20:8251940 in positive strand) and PLCB1-IT1 (chr20: 8251936 in positive strand)
3	Integration sites 10-100 bp pair apart (10-100 bp stagger)	EIF4G3 (chr1:20971026 in negative strand and EIF4G3 (chr1:20971114 in positive strand)
4	Integration sites 100 - 3000 bp apart (100-3000 bp stagger)	KATNA1(chr6: 149598014 in negative strand and KATNA1 (chr6: 149596202 in positive strand)

b

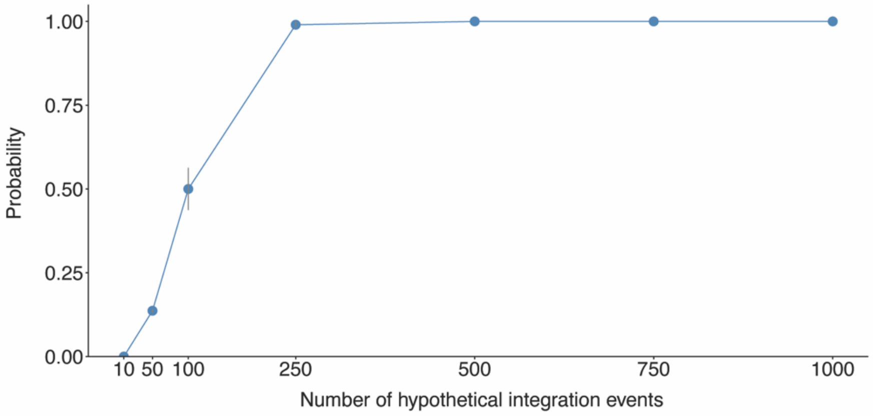

Figure S5. Identification of look-alike integration site artifact cases and the relationship between integration events and the probability of having true multiple integrations into the same gene(s). (a) Characterization of artifacts generated during the integration sites assay. Each staggered case is distinguished based on its genomic distance (bp) from the high-confidence integration sites coordinates of a putative clone. The examples shown here were observed in distinct putative clones. Bp: base pairs. **(b)** Regarding Step 3 in our proposed quality filter which involves collapsing integration sites that mapped to the same gene if found within a single reaction: We recognize that when PRISM-seq is applied to non-clonal clinical samples, there will be a genuine possibility that proviruses had integrated into distinct locations within the same gene. The likelihood of detecting genuine gene integration depends on the number of integration events sampled within a PRISM-seq reaction. We performed a Monte Carlo simulation and confirmed that the likelihood of detecting genuine same-gene integration was 0%, 0%, 14%, 50% and 99% when 1, 10, 50, 100, and 250 unique integration events were sampled. Therefore, we suggest that users should quantify targeting viral DNA copies by techniques such as Droplet Digital PCR (ddPCR), and turn off Step 3 if more than 50 target copies were input into each PRISM-seq reaction, with the caveat that specificity would be compromised. In this study, all reactions were performed at 50 copies per reaction input.

Figure S6

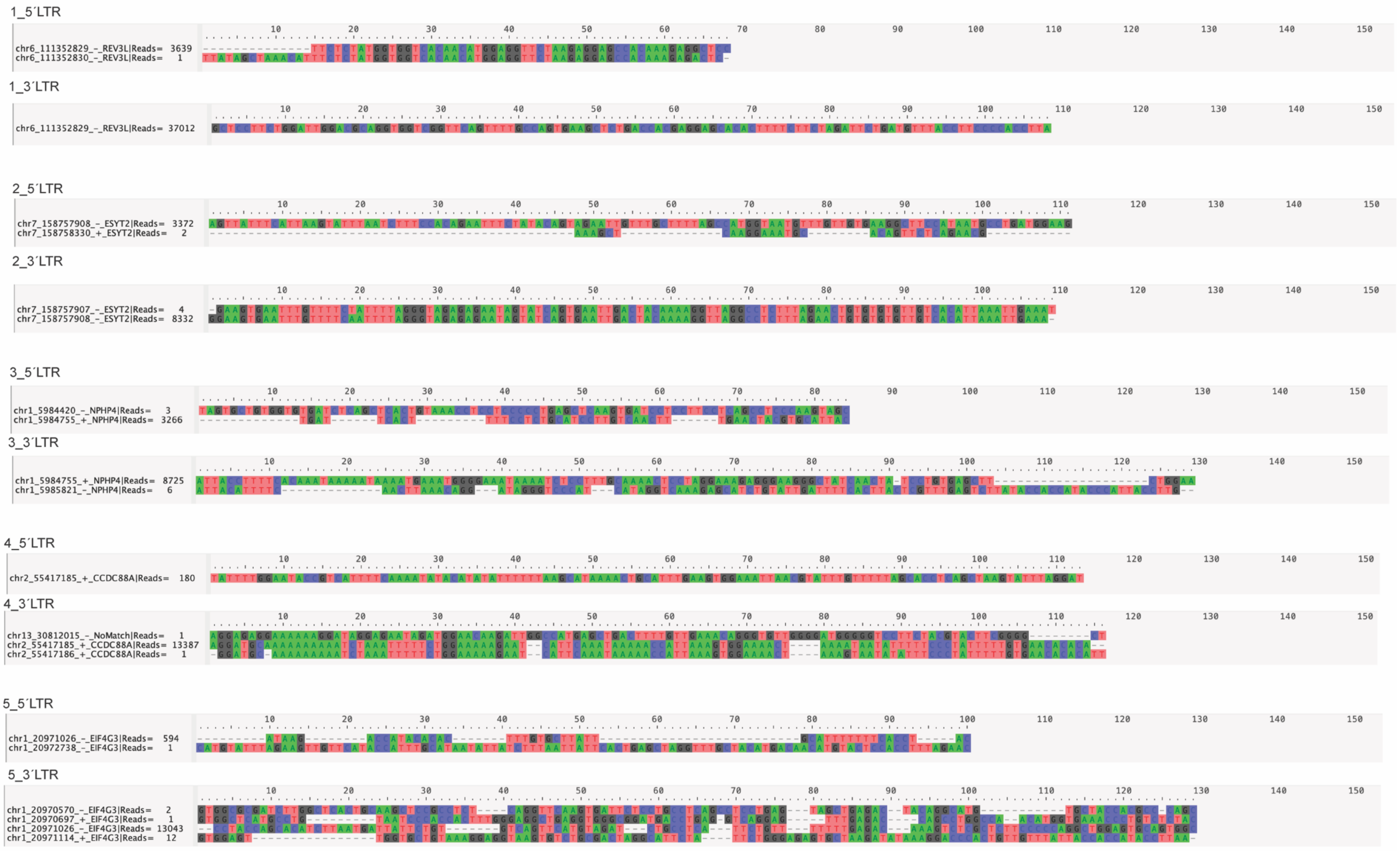

1_5´LTR
chr6_111352829_-_REV3L|Reads= 3639
chr6_111352830_-_REV3L|Reads= 1
1_3´LTR
chr6_111352829_-_REV3L|Reads= 37012
2_5´LTR
chr7_158757908_-_ESYT2|Reads= 3372
chr7_158758330_+_ESYT2|Reads= 2
2_3´LTR
chr7_158757907_-_ESYT2|Reads= 4
chr7_158757908_-_ESYT2|Reads= 8332
3_5´LTR
chr1_5984420_-_NPHP4|Reads= 3
chr1_5984755_+_NPHP4|Reads= 3266
3_3´LTR
chr1_5984755_+_NPHP4|Reads= 8725
chr1_5985821_-_NPHP4|Reads= 6
4_5´LTR
chr2_55417185_+_CCDC88A|Reads= 180
4_3´LTR
chr13_30812015_-_NoMatch|Reads= 1
chr2_55417185_+_CCDC88A|Reads= 13387
chr2_55417186_+_CCDC88A|Reads= 1
5_5´LTR
chr1_20971026_-_EIF4G3|Reads= 594
chr1_20972738_-_EIF4G3|Reads= 1
5_3´LTR
chr1_20970570_-_EIF4G3|Reads= 2
chr1_20970697_+_EIF4G3|Reads= 1
chr1_20971026_-_EIF4G3|Reads= 13043
chr1_20971114_+_EIF4G3|Reads= 12

Figure S6. Comparison of host sequences for each unique integration site found within the putative clones. The host sequence derived from the viral-host chimeric junction read for individual integration sites retrieved from putative clones 1-5 by the 5′LTR and 3′LTR viral-host junction. The low-confidence integration sites share the same junction sequence as the high-confidence integration site for clones 1, 2, and 4.

Figure S7

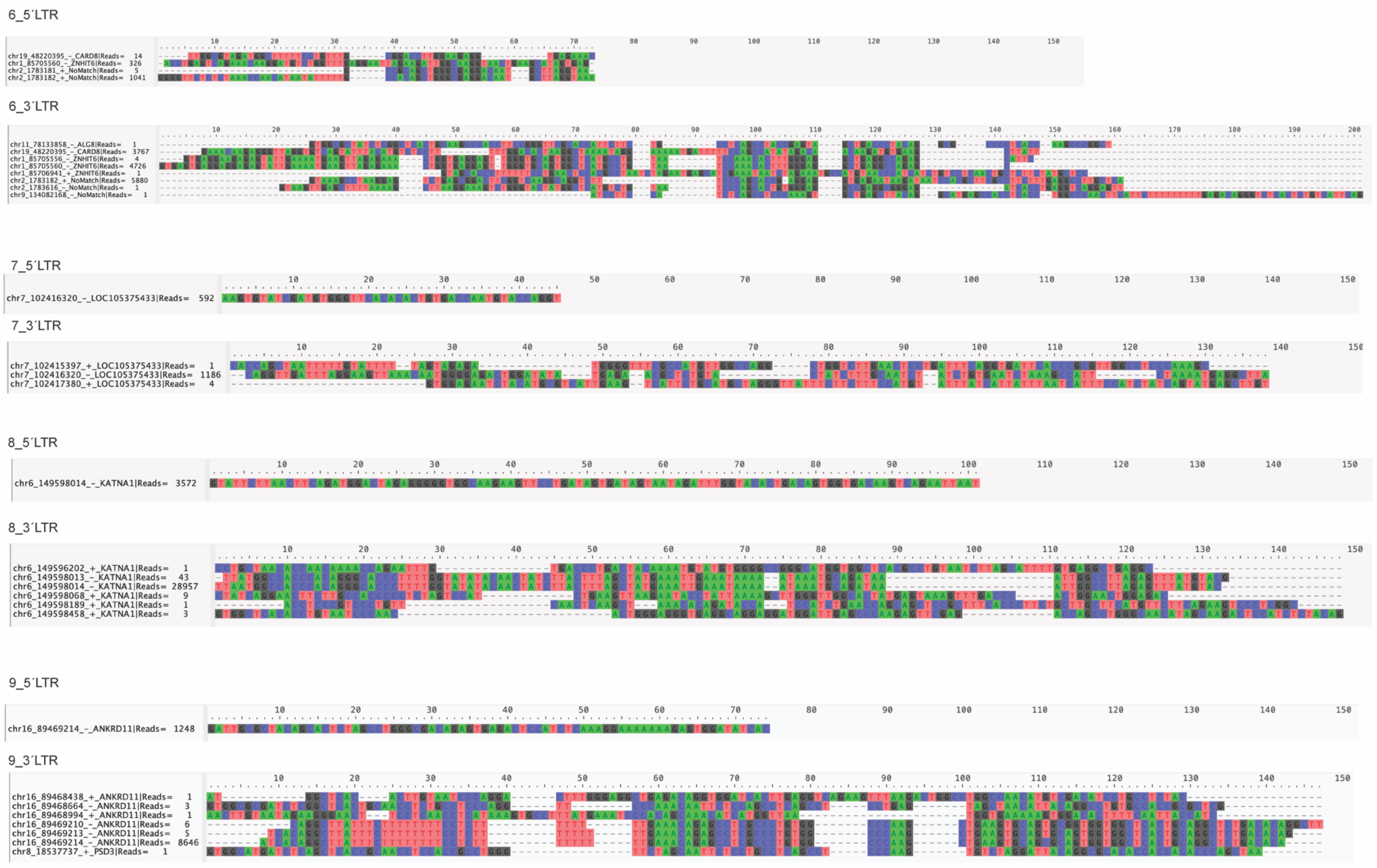
6_5´LTR
chr19_48220395_-_CARD8|Reads= 14
chr1_85705560_-_ZNHIT6|Reads= 326
chr2_1783181_+_NoMatch|Reads= 5
chr2_1783182_+_NoMatch|Reads= 1041
6_3´LTR
chr11_78133858_-_ALG8|Reads= 1
chr19_48220395_-_CARD8|Reads= 3767
chr1_85705556_-_ZNHIT6|Reads= 4
chr1_85705560_-_ZNHIT6|Reads= 4726
chr1_85706941_+_ZNHIT6|Reads= 1
chr2_1783182_+_NoMatch|Reads= 5880
chr2_1783616_-_NoMatch|Reads= 1
chr9_134082168_-_NoMatch|Reads= 1
7_5´LTR
chr7_102416320_-_LOC105375433|Reads= 592
7_3´LTR
chr7_102415397_+_LOC105375433|Reads= 1
chr7_102416320_-_LOC105375433|Reads= 1186
chr7_102417380_+_LOC105375433|Reads= 4
8_5´LTR
chr6_149598014_-_KATNA1|Reads= 3572
8_3´LTR
chr6_149596202_+_KATNA1|Reads= 1
chr6_149598013_-_KATNA1|Reads= 43
chr6_149598014_-_KATNA1|Reads= 28957
chr6_149598068_+_KATNA1|Reads= 9
chr6_149598189_+_KATNA1|Reads= 1
chr6_149598458_+_KATNA1|Reads= 3
9_5´LTR
chr16_89469214_-_ANKRD11|Reads= 1248
9_3´LTR
chr16_89468438_+_ANKRD11|Reads= 1
chr16_89468664_-_ANKRD11|Reads= 3
chr16_89468994_+_ANKRD11|Reads= 1
chr16_89469210_-_ANKRD11|Reads= 6
chr16_89469213_-_ANKRD11|Reads= 5
chr16_89469214_-_ANKRD11|Reads= 8646
chr8_18537737_+_PSD3|Reads= 1

Figure S7. Comparison of host sequences for each unique integration site found within the putative clones. The host sequence derived from the viral-host chimeric junction read for individual integration sites retrieved from putative clones 6-9 by the 5′LTR and 3′LTR viral-host junction. The low-confidence integration sites share the same junction sequence as the high-confidence integration site for clones 6, 8, and 9.

Figure S8

10_5´LTR

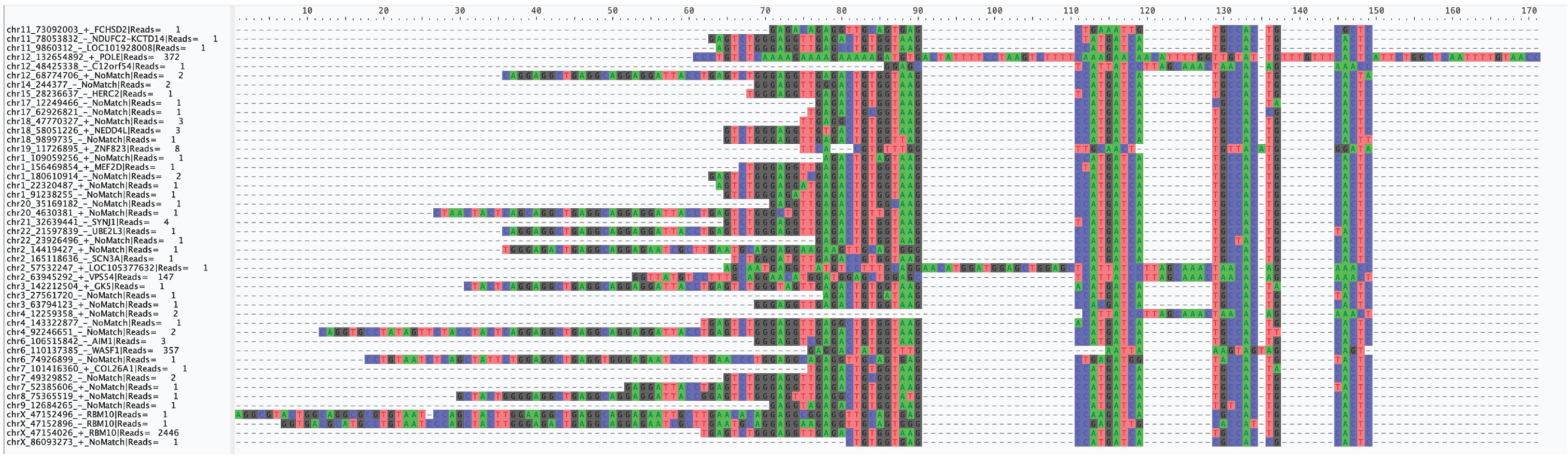

10_3´LTR

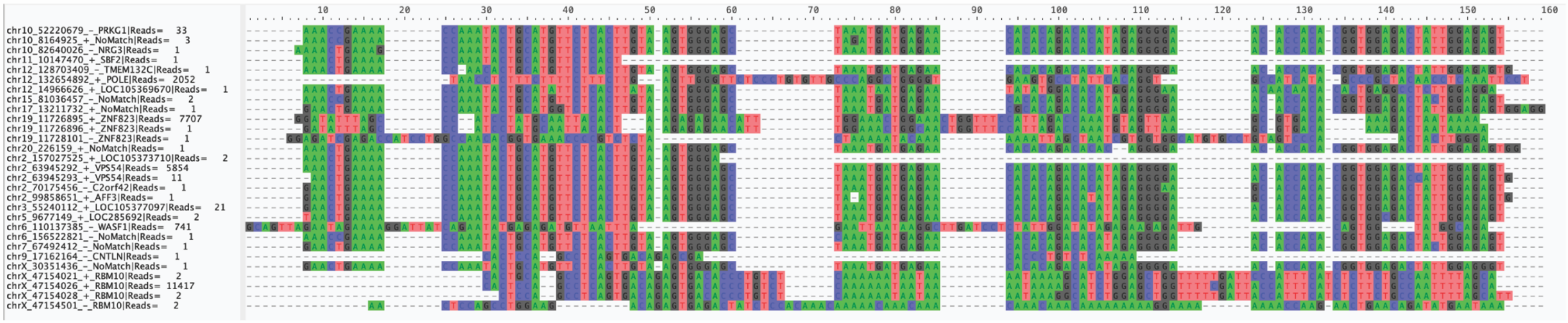

Figure S8. Comparison of host sequences for each unique integration site found within the putative clones. The host sequence derived from the viral-host chimeric junction read for individual integration sites retrieved from putative clone 10 by the 5′LTR and 3′LTR viral-host junction. The low-confidence integration sites share the same junction sequence as the high-confidence integration site for clone 10.

Figure S9

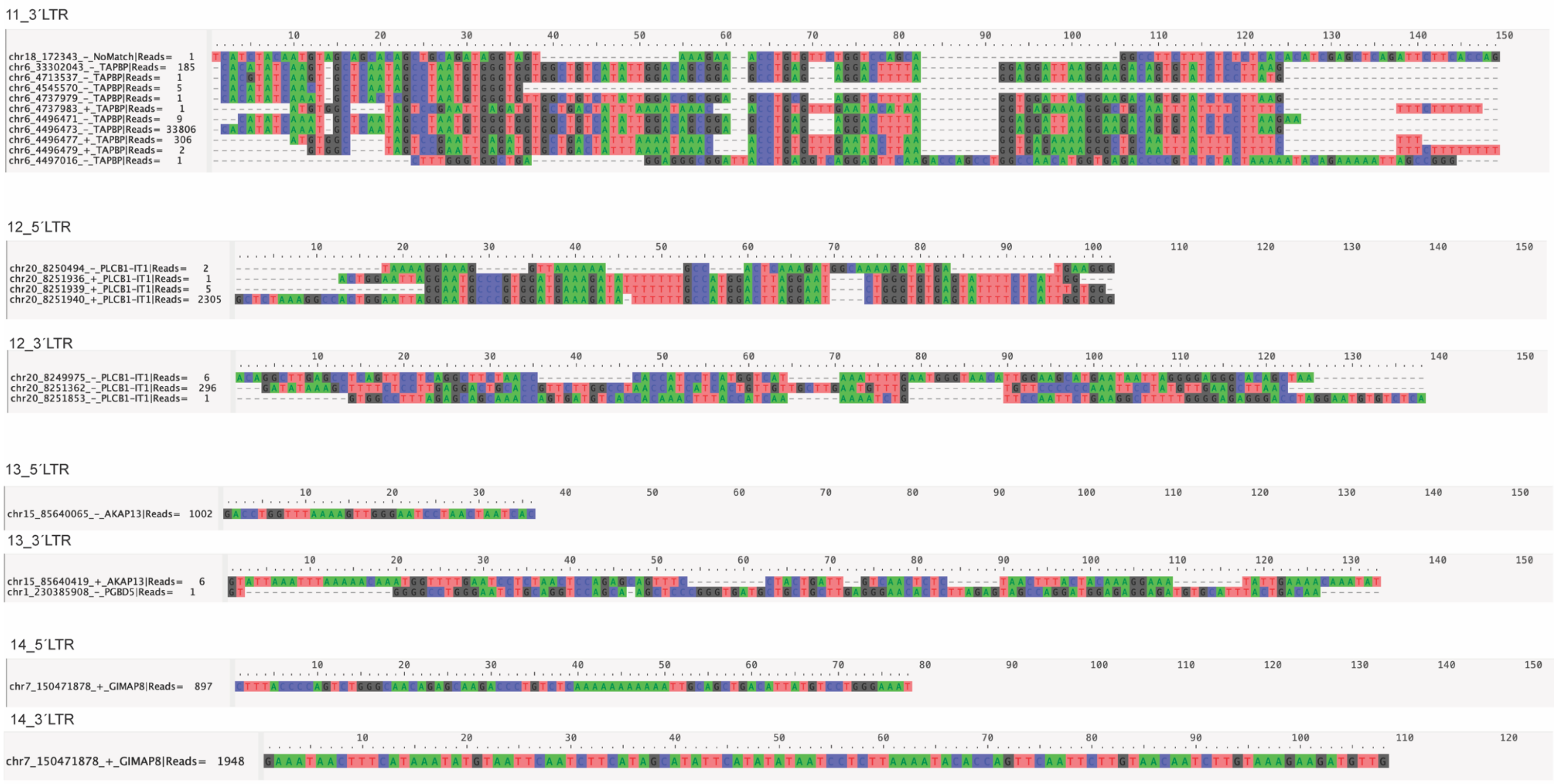
11_3´LTR
chr18_172343_-_NoMatch|Reads= 1
chr6_33302043_-_TAPBP|Reads= 185
chr6_4713537_-_TAPBP|Reads= 1
chr6_4545570_-_TAPBP|Reads= 5
chr6_4737979_-_TAPBP|Reads= 1
chr6_4737983_+_TAPBP|Reads= 1
chr6_4496471_-_TAPBP|Reads= 9
chr6_4496473_-_TAPBP|Reads= 33806
chr6_4496477_+_TAPBP|Reads= 306
chr6_4496479_+_TAPBP|Reads= 2
chr6_4497016_-_TAPBP|Reads= 1
12_5´LTR
chr20_8250494_-_PLCB1-IT1|Reads= 2
chr20_8251936_+_PLCB1-IT1|Reads= 1
chr20_8251939_+_PLCB1-IT1|Reads= 5
chr20_8251940_+_PLCB1-IT1|Reads= 2305
12_3´LTR
chr20_8249975_-_PLCB1-IT1|Reads= 6
chr20_8251362_-_PLCB1-IT1|Reads= 296
chr20_8251853_-_PLCB1-IT1|Reads= 1
13_5´LTR
chr15_85640065_-_AKAP13|Reads= 1002
13_3´LTR
chr15_85640419_+_AKAP13|Reads= 6
chr1_230385908_-_PGBD5|Reads= 1
14_5´LTR
chr7_150471878_+_GIMAP8|Reads= 897
14_3´LTR
chr7_150471878_+_GIMAP8|Reads= 1948

Figure S9. Comparison of host sequences for each unique integration site found within the putative clones. The host sequence derived from the viral-host chimeric junction read for individual integration site retrieved from putative clones 11-14 by the 5′LTR and 3′LTR viral-host junction. The low-confidence integration sites share the same junction sequence as the high-confidence integration site for clones 11 and 12.

Figure S10

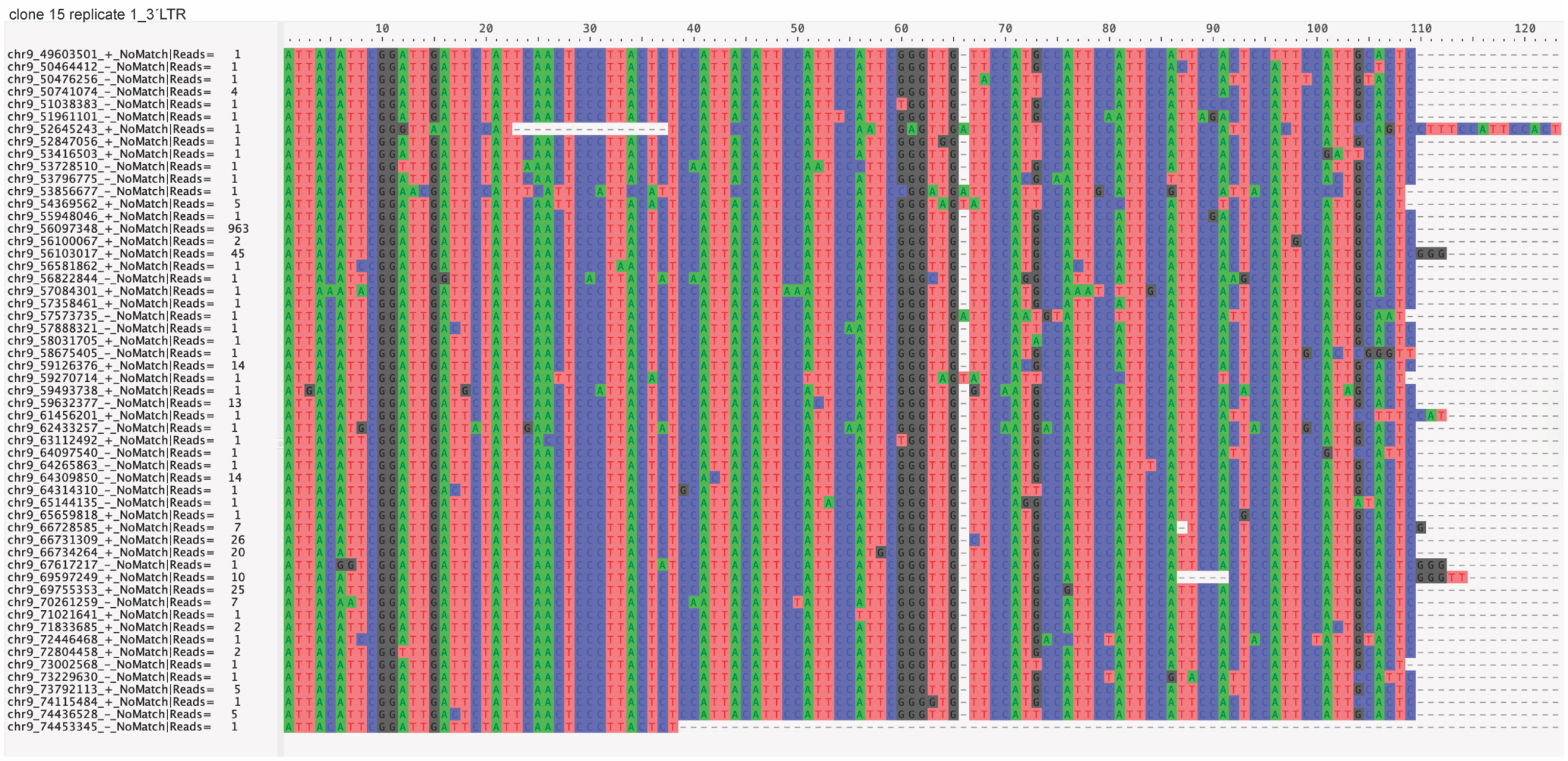
clone 15 replicate 1_3´LTR
10
20
30
40
50
60
70
80
90
100
110
120
chr9_49603501_+_NoMatch|Reads= 1
chr9_50464412_-_NoMatch|Reads= 1
chr9_50476256_-_NoMatch|Reads= 1
chr9_50741074_-_NoMatch|Reads= 4
chr9_51038383_-_NoMatch|Reads= 1
chr9_51961101_-_NoMatch|Reads= 1
chr9_52645243_+_NoMatch|Reads= 1
chr9_52847056_+_NoMatch|Reads= 1
chr9_53416503_+_NoMatch|Reads= 1
chr9_53728510_-_NoMatch|Reads= 1
chr9_53796775_-_NoMatch|Reads= 1
chr9_53856677_-_NoMatch|Reads= 1
chr9_54369562_+_NoMatch|Reads= 5
chr9_55948046_+_NoMatch|Reads= 1
chr9_56097348_+_NoMatch|Reads= 963
chr9_56100067_+_NoMatch|Reads= 2
chr9_56103017_+_NoMatch|Reads= 45
chr9_56581862_+_NoMatch|Reads= 1
chr9_56822844_-_NoMatch|Reads= 1
chr9_57084301_+_NoMatch|Reads= 1
chr9_57358461_+_NoMatch|Reads= 1
chr9_57573735_-_NoMatch|Reads= 1
chr9_57888321_-_NoMatch|Reads= 1
chr9_58031705_+_NoMatch|Reads= 1
chr9_58675405_-_NoMatch|Reads= 1
chr9_59126376_+_NoMatch|Reads= 14
chr9_59270714_+_NoMatch|Reads= 1
chr9_59493738_+_NoMatch|Reads= 1
chr9_59632377_-_NoMatch|Reads= 13
chr9_61456201_+_NoMatch|Reads= 1
chr9_62433257_-_NoMatch|Reads= 1
chr9_63112492_+_NoMatch|Reads= 1
chr9_64097540_-_NoMatch|Reads= 1
chr9_64265863_-_NoMatch|Reads= 1
chr9_64309850_-_NoMatch|Reads= 14
chr9_64314310_-_NoMatch|Reads= 1
chr9_65144135_-_NoMatch|Reads= 1
chr9_65659818_+_NoMatch|Reads= 1
chr9_66728585_+_NoMatch|Reads= 7
chr9_66731309_+_NoMatch|Reads= 26
chr9_66734264_+_NoMatch|Reads= 20
chr9_67617217_-_NoMatch|Reads= 1
chr9_69597249_+_NoMatch|Reads= 10
chr9_69755353_+_NoMatch|Reads= 25
chr9_70261259_-_NoMatch|Reads= 7
chr9_71021641_+_NoMatch|Reads= 1
chr9_71833685_+_NoMatch|Reads= 2
chr9_72446468_+_NoMatch|Reads= 1
chr9_72804458_+_NoMatch|Reads= 2
chr9_73002568_-_NoMatch|Reads= 1
chr9_73229630_-_NoMatch|Reads= 1
chr9_73792113_+_NoMatch|Reads= 5
chr9_74115484_+_NoMatch|Reads= 1
chr9_74436528_-_NoMatch|Reads= 5
chr9_74453345_-_NoMatch|Reads= 1

Figure S10. Comparison of host sequences for each unique integration site found in clones isolated from HIV-1-infected individuals. The host sequence derived from the 3′ LTR viral-host chimeric junction read for all unique integration sites identified for clone #15 replicate 1. The low-confidence integration sites share the same junction sequence as the high-confidence integration site for clone 15.

Figure S11

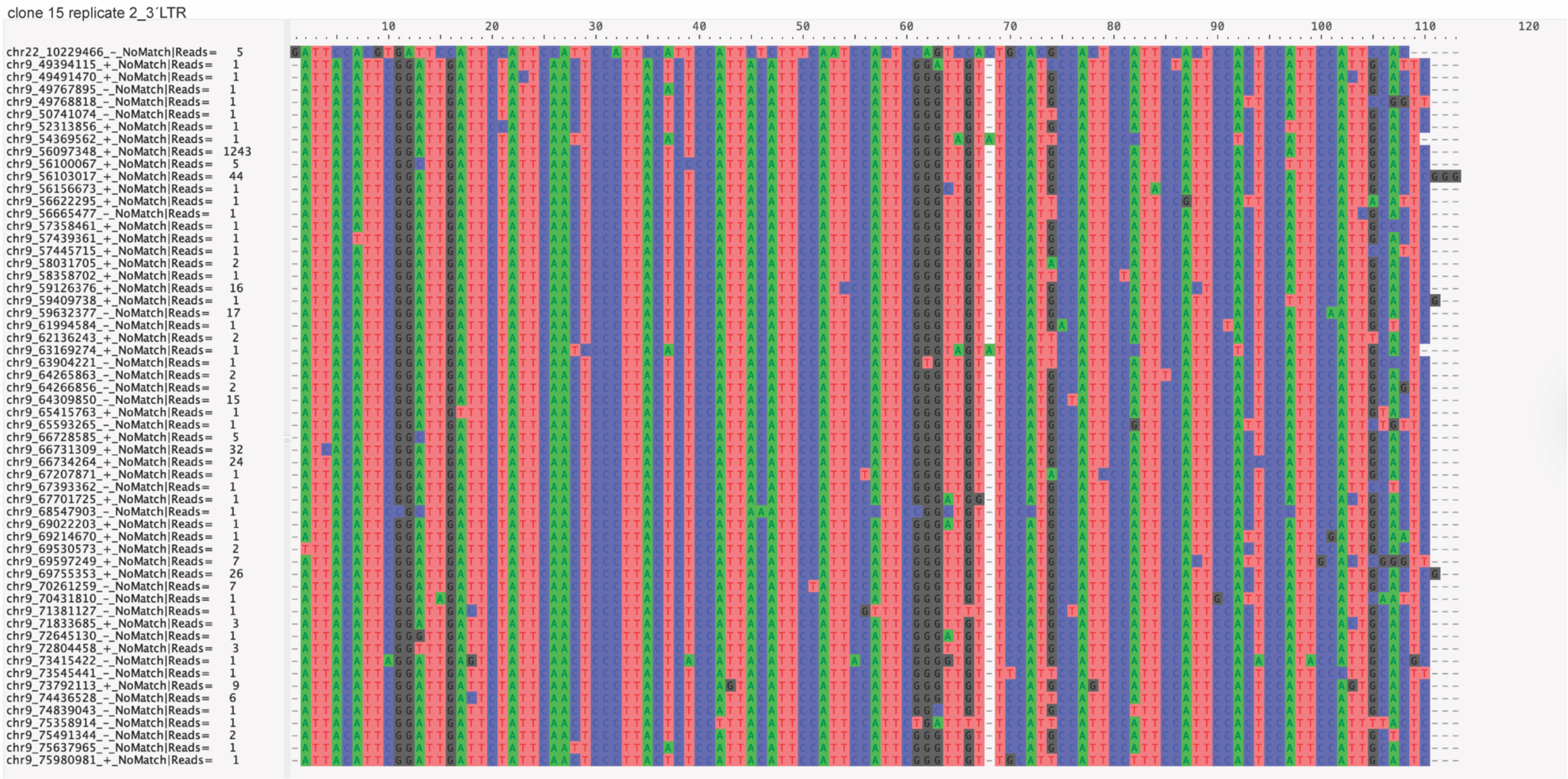
clone 15 replicate 2_3´LTR
10 20 30 40 50 60 70 80 90 100 110 120
chr22_10229466_-_NoMatch|Reads= 5
chr9_49394115_+_NoMatch|Reads= 1
chr9_49491470_+_NoMatch|Reads= 1
chr9_49767895_-_NoMatch|Reads= 1
chr9_49768818_-_NoMatch|Reads= 1
chr9_50741074_-_NoMatch|Reads= 1
chr9_52313856_+_NoMatch|Reads= 1
chr9_54369562_+_NoMatch|Reads= 1
chr9_56097348_+_NoMatch|Reads= 1243
chr9_56100067_+_NoMatch|Reads= 5
chr9_56103017_+_NoMatch|Reads= 44
chr9_56156673_+_NoMatch|Reads= 1
chr9_56622295_+_NoMatch|Reads= 1
chr9_56665477_-_NoMatch|Reads= 1
chr9_57358461_+_NoMatch|Reads= 1
chr9_57439361_+_NoMatch|Reads= 1
chr9_57445715_+_NoMatch|Reads= 1
chr9_58031705_+_NoMatch|Reads= 2
chr9_58358702_+_NoMatch|Reads= 1
chr9_59126376_+_NoMatch|Reads= 16
chr9_59409738_+_NoMatch|Reads= 1
chr9_59632377_-_NoMatch|Reads= 17
chr9_61994584_-_NoMatch|Reads= 1
chr9_62136243_+_NoMatch|Reads= 2
chr9_63169274_+_NoMatch|Reads= 1
chr9_63904221_-_NoMatch|Reads= 1
chr9_64265863_-_NoMatch|Reads= 2
chr9_64266856_-_NoMatch|Reads= 2
chr9_64309850_-_NoMatch|Reads= 15
chr9_65415763_+_NoMatch|Reads= 1
chr9_65593265_-_NoMatch|Reads= 1
chr9_66728585_+_NoMatch|Reads= 5
chr9_66731309_+_NoMatch|Reads= 32
chr9_66734264_+_NoMatch|Reads= 24
chr9_67207871_+_NoMatch|Reads= 1
chr9_67393362_-_NoMatch|Reads= 1
chr9_67701725_+_NoMatch|Reads= 1
chr9_68547903_-_NoMatch|Reads= 1
chr9_69022203_+_NoMatch|Reads= 1
chr9_69214670_+_NoMatch|Reads= 1
chr9_69530573_+_NoMatch|Reads= 2
chr9_69597249_+_NoMatch|Reads= 7
chr9_69755353_+_NoMatch|Reads= 26
chr9_70261259_-_NoMatch|Reads= 7
chr9_70431810_-_NoMatch|Reads= 1
chr9_71381127_-_NoMatch|Reads= 1
chr9_71833685_+_NoMatch|Reads= 3
chr9_72645130_-_NoMatch|Reads= 1
chr9_72804458_+_NoMatch|Reads= 3
chr9_73415422_-_NoMatch|Reads= 1
chr9_73545441_-_NoMatch|Reads= 1
chr9_73792113_+_NoMatch|Reads= 9
chr9_74436528_-_NoMatch|Reads= 6
chr9_74839043_-_NoMatch|Reads= 1
chr9_75358914_-_NoMatch|Reads= 1
chr9_75491344_-_NoMatch|Reads= 2
chr9_75637965_-_NoMatch|Reads= 1
chr9_75980981_+_NoMatch|Reads= 1

Figure S11. Comparison of host sequences for each unique integration site found in clones isolated from HIV-1-infected individuals. The host sequence derived from the 3′ LTR viral-host chimeric junction read for all unique integration sites identified for clone #15 replicate 2. The low-confidence integration sites share the same junction sequence as the high-confidence integration site for clone 15.

Figure S12

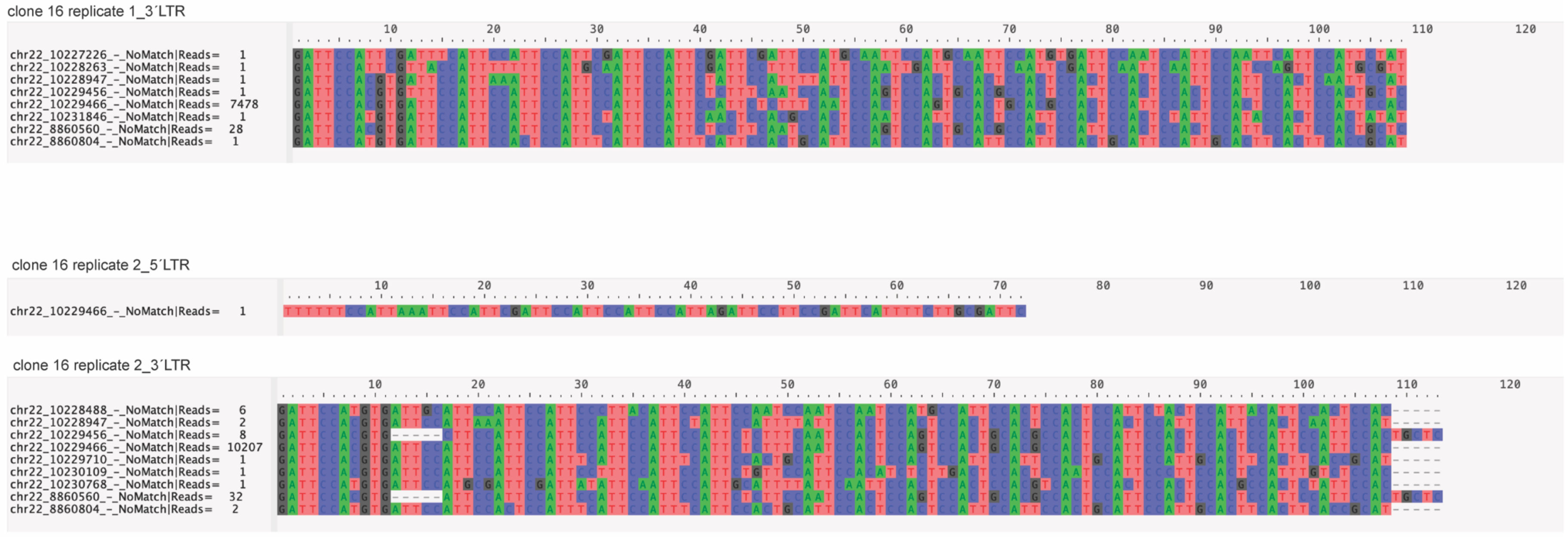

clone 16 replicate 1_3´LTR
chr22_10227226_-_NoMatch|Reads= 1
chr22_10228263_-_NoMatch|Reads= 1
chr22_10228947_-_NoMatch|Reads= 1
chr22_10229456_-_NoMatch|Reads= 1
chr22_10229466_-_NoMatch|Reads= 7478
chr22_10231846_-_NoMatch|Reads= 1
chr22_8860560_-_NoMatch|Reads= 28
chr22_8860804_-_NoMatch|Reads= 1
clone 16 replicate 2_5´LTR
chr22_10229466_-_NoMatch|Reads= 1
clone 16 replicate 2_3´LTR
chr22_10228488_-_NoMatch|Reads= 6
chr22_10228947_-_NoMatch|Reads= 2
chr22_10229456_-_NoMatch|Reads= 8
chr22_10229466_-_NoMatch|Reads= 10207
chr22_10229710_-_NoMatch|Reads= 1
chr22_10230109_-_NoMatch|Reads= 1
chr22_10230768_-_NoMatch|Reads= 1
chr22_8860560_-_NoMatch|Reads= 32
chr22_8860804_-_NoMatch|Reads= 2

Figure S12. Comparison of host sequences for each unique integration site found in clones isolated from HIV-1-infected individuals. The host sequence derived from the viral-host chimeric junction read for all unique integration sites identified for clone #16 replicates 1 and 2, respectively. The low-confidence integration sites share the same junction sequence as the high-confidence integration site for clone 16.

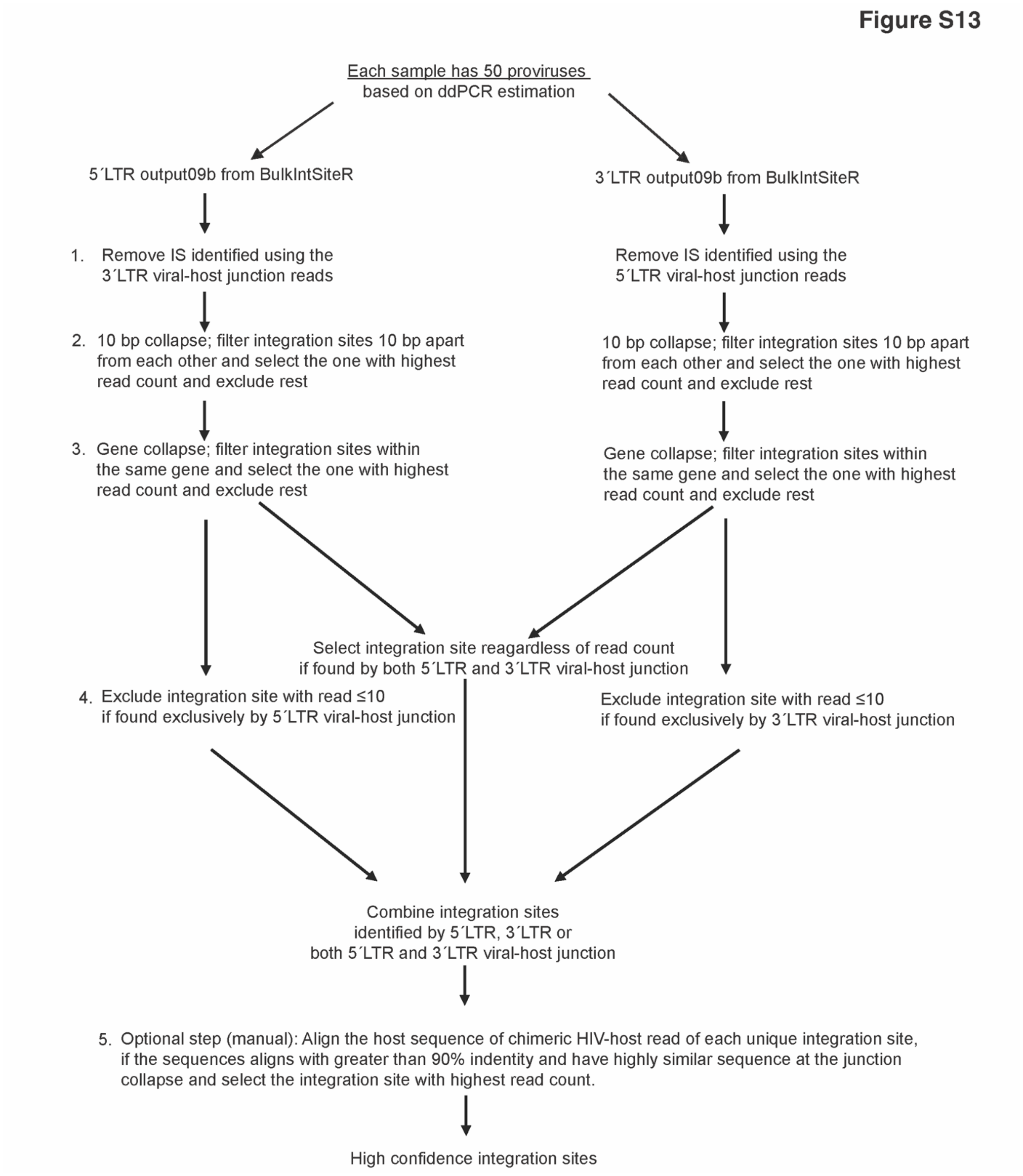

Figure S13. Data-guided noise removal approach. Leveraging the clonal nature of Clones #1-16, we devised a final quality control filter to remove assay noise. This set of quality filters is applied to the BulkIntSiteR output; (1) filter and remove the spillover noise of integration site identified by 3′ LTR in the 5′ LTR library and vice versa. (2) integration sites within ≤10 bp were collapsed by retaining the site with the highest read count; (3) integration sites located within the same gene were filtered to retain only one site with the highest read count, excluding all others if the input template count is ≤50 copies; (4) integration sites detected exclusively by either 5′ LTR or 3′ LTR junctions were excluded if supported by ≤10 read counts. Integration sites detected by

both 5′ LTR and 3′ LTR virus-host junctions, regardless of the raw read counts, and the sites that passed step 4 were classified as high-confidence. An optional Step (5), involving user-guided homology clustering of host sequences derived from the viral-host chimeric reads, removes look-alike integration sites that share identical junction sequences and co-occur within the same reaction, retaining only the site with the highest read count (Fig. S9). Steps 1-4 are fully automated, and the accompanying script is included with the BulkIntSiteR package; Step 5 requires user input and manual curation. If Step 5 is not performed, PRISM-seq results in 5.7% (10/174) noise in our data. This final step 5 is particularly critical when proviruses are expected to be integrated within repeat-rich genomic regions. Without Step 5, BulkIntSiteR identified 7 of 8 (87.5%) and 1 of 2 (50%) integration sites as low-confidence (noise) for the two clones (#15 and #16) with provirus integrated in centromeric regions (Fig. S14a-e). Incorporating step 5 and removing redundant low-confidence sites sharing the same junction sequence yielded one high-confidence integration site for each clone (0% noise), underscoring its importance for accurate proviral insertion site detection in repetitive loci.

Figure S14

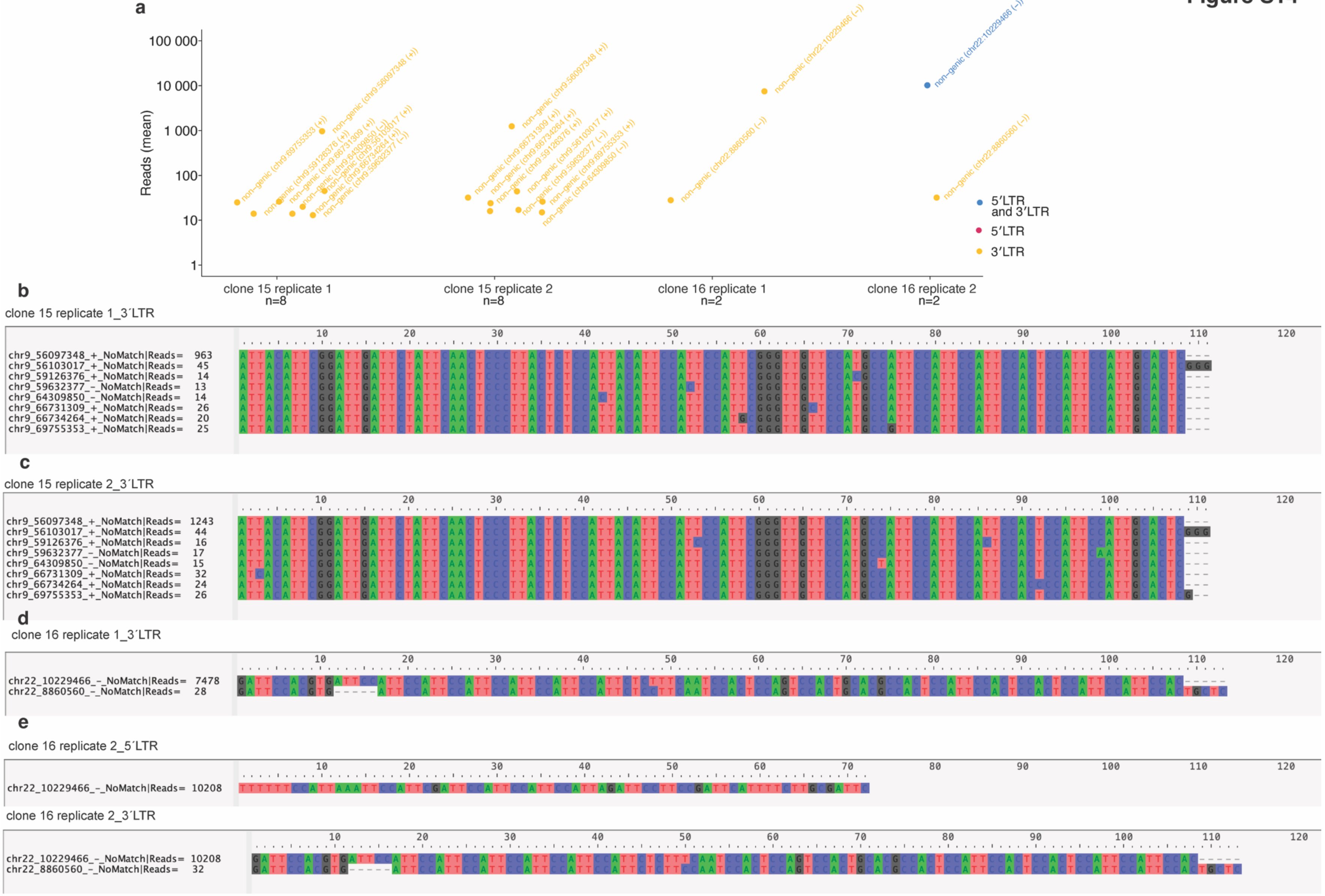

a
Reads (mean)
100 000
10 000
1 000
100
10
1
clone 15 replicate 1
n=8
clone 15 replicate 2
n=8
clone 16 replicate 1
n=2
clone 16 replicate 2
n=2
5′LTR and 3′LTR
5′LTR
3′LTR
b
clone 15 replicate 1_3′LTR
chr9_56097348_+_NoMatch|Reads= 963
chr9_56103017_+_NoMatch|Reads= 45
chr9_59126376_+_NoMatch|Reads= 14
chr9_59632377_-_NoMatch|Reads= 13
chr9_64309850_-_NoMatch|Reads= 14
chr9_66731309_+_NoMatch|Reads= 26
chr9_66734264_+_NoMatch|Reads= 20
chr9_69755353_+_NoMatch|Reads= 25
c
clone 15 replicate 2_3′LTR
chr9_56097348_+_NoMatch|Reads= 1243
chr9_56103017_+_NoMatch|Reads= 44
chr9_59126376_+_NoMatch|Reads= 16
chr9_59632377_-_NoMatch|Reads= 17
chr9_64309850_-_NoMatch|Reads= 15
chr9_66731309_+_NoMatch|Reads= 32
chr9_66734264_+_NoMatch|Reads= 24
chr9_69755353_+_NoMatch|Reads= 26
d
clone 16 replicate 1_3′LTR
chr22_10229466_-_NoMatch|Reads= 7478
chr22_8860560_-_NoMatch|Reads= 28
e
clone 16 replicate 2_5′LTR
chr22_10229466_-_NoMatch|Reads= 10208
clone 16 replicate 2_3′LTR
chr22_10229466_-_NoMatch|Reads= 10208
chr22_8860560_-_NoMatch|Reads= 32

Figure S14. Post-filter recovery of proviral integration sites found within centromeric clones. **(a)** The BulkIntSiteR integration sites output data was processed using the QC filtering pipeline, and results for clones #15 and #16 are shown for technical duplicate reactions. **(b-e)** Comparison of the host sequence for all unique integration sites after QC filtering for clones #15 and #16. The unexpected low-confidence integration sites share the same junction sequence as the high-confidence integration site for clones 15 and 16.

Figure S15

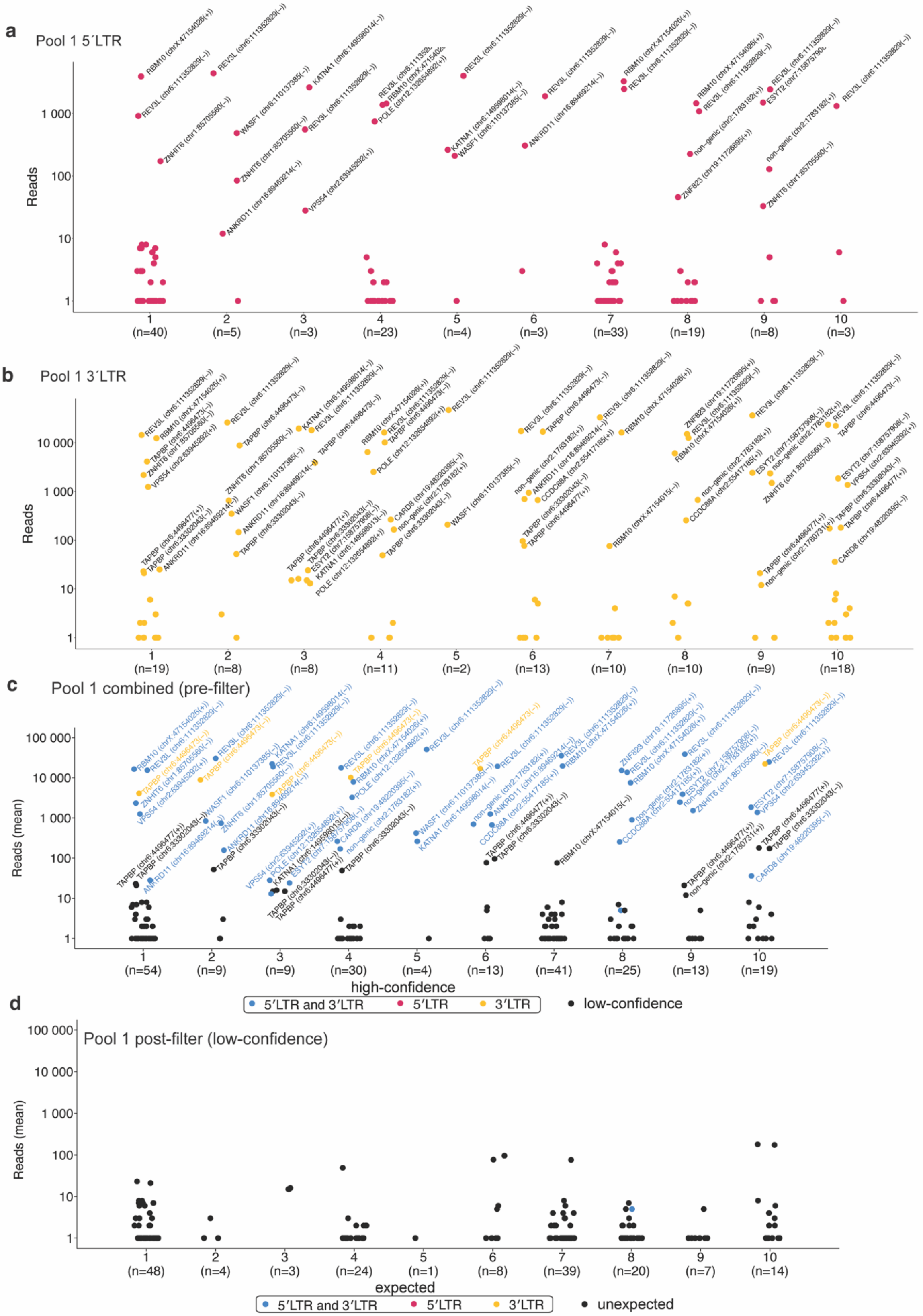

a
Pool 1 5′LTR
Reads
1
10
100
1 000
1 (n=40)
2 (n=5)
3 (n=3)
4 (n=23)
5 (n=4)
6 (n=3)
7 (n=33)
8 (n=19)
9 (n=8)
10 (n=3)
b
Pool 1 3′LTR
Reads
1
10
100
1 000
10 000
1 (n=19)
2 (n=8)
3 (n=8)
4 (n=11)
5 (n=2)
6 (n=13)
7 (n=10)
8 (n=10)
9 (n=9)
10 (n=18)
c
Pool 1 combined (pre-filter)
Reads (mean)
1
10
100
1 000
10 000
100 000
1 (n=54)
2 (n=9)
3 (n=9)
4 (n=30)
5 (n=4)
6 (n=13)
7 (n=41)
8 (n=25)
9 (n=13)
10 (n=19)
high-confidence
5′LTR and 3′LTR
5′LTR
3′LTR
low-confidence
d
Pool 1 post-filter (low-confidence)
Reads (mean)
1
10
100
1 000
10 000
100 000
1 (n=48)
2 (n=4)
3 (n=3)
4 (n=24)
5 (n=1)
6 (n=8)
7 (n=39)
8 (n=20)
9 (n=7)
10 (n=14)
expected
5′LTR and 3′LTR
5′LTR
3′LTR
unexpected

Figure S15. Proviral integration sites identification in artificial pool 1. (a-c) The dot plot represents the total number of unique integration sites identified in each replicate of pool 1 by either the 5′LTR (a) or 3′LTR (b) or combined (c) viral-host junction. The total number of unique integration sites retrieved in an individual replicate is represented as an n value. The blue, red, and yellow dots represent the integration site identified by both 5′LTR and 3′LTR, exclusively by 5′LTR or 3′LTR, respectively. (d) Post-filter identification of low-confidence expected and unexpected integration sites.

Figure S16

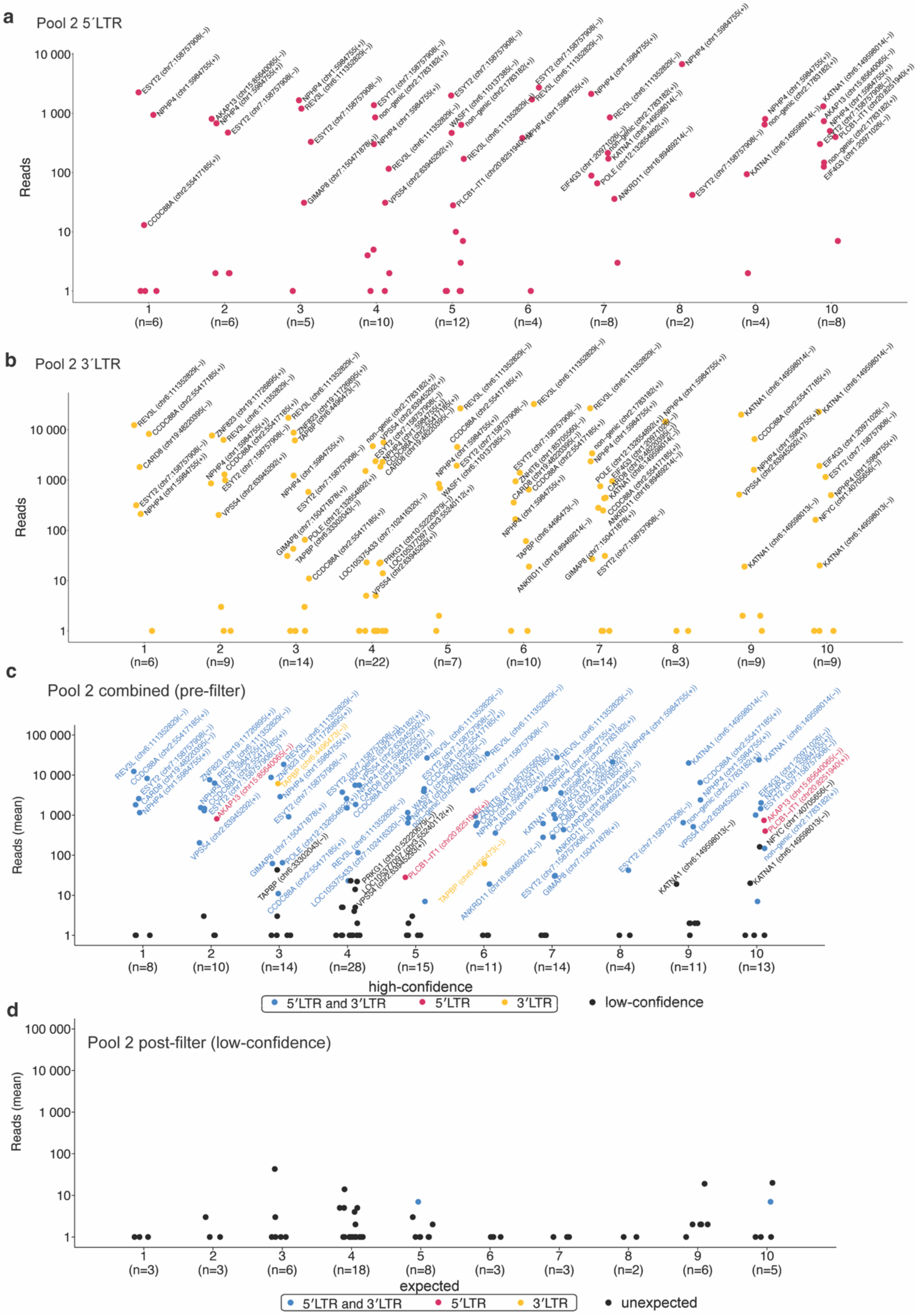
a
Pool 2 5´LTR
Reads
10 000
1 000
100
10
1
1 (n=6)
2 (n=6)
3 (n=5)
4 (n=10)
5 (n=12)
6 (n=4)
7 (n=8)
8 (n=2)
9 (n=4)
10 (n=8)
b
Pool 2 3´LTR
Reads
10 000
1 000
100
10
1
1 (n=6)
2 (n=9)
3 (n=14)
4 (n=22)
5 (n=7)
6 (n=10)
7 (n=14)
8 (n=3)
9 (n=9)
10 (n=9)
c
Pool 2 combined (pre-filter)
Reads (mean)
100 000
10 000
1 000
100
10
1
1 (n=8)
2 (n=10)
3 (n=14)
4 (n=28)
5 (n=15)
6 (n=11)
7 (n=14)
8 (n=4)
9 (n=11)
10 (n=13)
high-confidence
5′LTR and 3′LTR
5′LTR
3′LTR
low-confidence
d
Pool 2 post-filter (low-confidence)
Reads (mean)
100 000
10 000
1 000
100
10
1
1 (n=3)
2 (n=3)
3 (n=6)
4 (n=18)
5 (n=8)
6 (n=3)
7 (n=3)
8 (n=2)
9 (n=6)
10 (n=5)
expected
5′LTR and 3′LTR
5′LTR
3′LTR
unexpected

Figure S16. Proviral integration sites identification in artificial pool 2. (a-c) The dot plot represents the total number of unique integration sites identified in each replicate of pool 2 by either the 5′LTR (a) or 3′LTR (b) or combined (c) viral-host junction. The total number of unique integration sites retrieved in an individual replicate is represented as an n value. The blue, red, and yellow dots represent the integration sites identified by both 5′LTR and 3′LTR, exclusively by 5′LTR or 3′LTR, respectively. (d) Post-filter identification of low-confidence expected and unexpected integration sites.

Figure S17

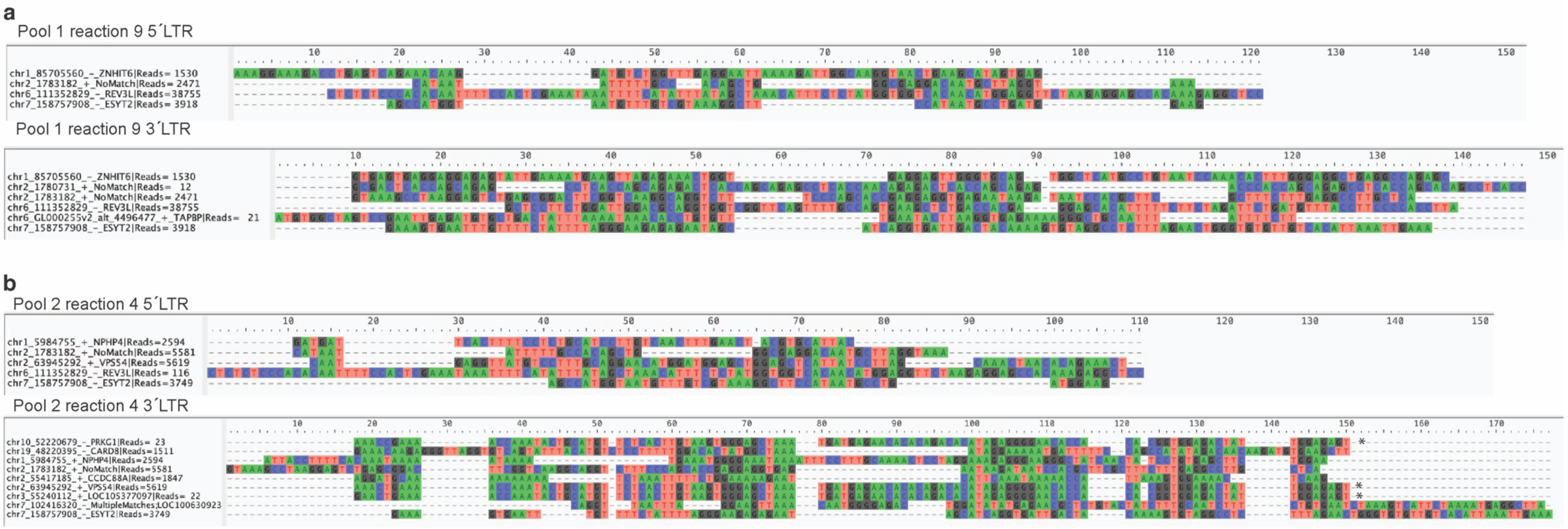
a
Pool 1 reaction 9 5´LTR
chr1_85705560_-_ZNHIT6|Reads= 1530
chr2_1783182_+_NoMatch|Reads= 2471
chr6_111352829_-_REV3L|Reads=38755
chr7_158757908_-_ESYT2|Reads= 3918
Pool 1 reaction 9 3´LTR
chr1_85705560_-_ZNHIT6|Reads= 1530
chr2_1780731_+_NoMatch|Reads= 12
chr2_1783182_+_NoMatch|Reads= 2471
chr6_111352829_-_REV3L|Reads=38755
chr6_GL000255v2_alt_4496477_+_TAPBP|Reads= 21
chr7_158757908_-_ESYT2|Reads= 3918
b
Pool 2 reaction 4 5´LTR
chr1_5984755_+_NPHP4|Reads=2594
chr2_1783182_+_NoMatch|Reads=5581
chr2_63945292_+_VPS54|Reads=5619
chr6_111352829_-_REV3L|Reads= 116
chr7_158757908_-_ESYT2|Reads=3749
Pool 2 reaction 4 3´LTR
chr10_52220679_-_PRKG1|Reads= 23
chr19_48220395_-_CARD8|Reads=1511
chr1_5984755_+_NPHP4|Reads=2594
chr2_1783182_+_NoMatch|Reads=5581
chr2_55417185_+_CCDC88A|Reads=1847
chr2_63945292_+_VPS54|Reads=5619
chr3_55240112_+_LOC105377097|Reads= 22
chr7_102416320_-_MultipleMatches;LOC100630923
chr7_158757908_-_ESYT2|Reads=3749

Figure S17. Comparison of host sequences of unexpected integration sites observed in pool 1 and pool 2, respectively (a and b). The host sequence derived from unexpected integration sites found in pool 1 replicate 9 (a) and pool 2 replicate 4 (b) by 5′LTR and 3′LTR viral-host junction. The unexpected integration sites sharing the same junction sequence or ±10 kb away from the expected high-confidence integration site are labelled with an asterisk (*).
